# Standardization and evaluation of indicators for quantifying antimicrobial use on U.S. dairy farms

**DOI:** 10.3389/frabi.2023.1176817

**Published:** 2023-08-14

**Authors:** Zhengyu Lu, Ece Bulut, Daryl V. Nydam, Renata Ivanek

**Affiliations:** ^1^ Department of Population Medicine and Diagnostic Sciences, College of Veterinary Medicine, Cornell University, Ithaca, NY, United States; ^2^ Department of Public and Ecosystem Health, College of Veterinary Medicine, Cornell University, Ithaca, NY, United States

**Keywords:** dairy cattle, antimicrobial use, antimicrobial stewardship, indicators, privacy

## Abstract

Antimicrobial resistance (AMR) is a global One Health threat. A portion of AMR development can be attributed to antimicrobial use (AMU) in animals, including dairy cattle. Quantifying AMU on U.S. dairy farms is necessary to inform antimicrobial stewardship strategies and help evaluate the relationship between AMU and AMR. Many AMU indicators have been proposed for quantifying AMU in dairy cattle. However, these indicators are difficult to interpret and compare because they differ in the type of data used, the calculation approach, and the definitions of variables and parameters used in the calculation. Therefore, we selected 16 indicators (count-based, mass-based, and dose-based) applicable for quantifying AMU on U.S. dairy farms. We systematized the indicators by standardizing their variables and parameters to improve their interchangeability, interpretation, and comparability. We scored indicators against six data-driven criteria (assessing their accuracy, data and effort needs, and level of privacy concern) and five stewardship-driven criteria (assessing their ability to capture trends and inform antimicrobial stewardship). The derived standardized indicators will aid farmers and veterinarians in selecting suitable indicators based on data availability and stewardship needs on a farm. The comparison of indicators revealed a trade-off requiring farmers to balance the granularity of data necessary for an accurate indicator and effort to collect the data, and a trade-off relevant to farmers interested in data sharing to inform stewardship because more accurate indicators are typically based on more sensitive information. Indicators with better accuracy tended to score better in stewardship criteria. Overall, two dose-based indicators, estimating the number of treatments and administered doses, scored best in accuracy and stewardship. Conversely, two count-based indicators, estimating the length of AMU, and a mass-based indicator, estimating the mass of administered antimicrobials, performed best in the effort and privacy criteria. These findings are expected to benefit One Health by aiding the uptake of farm-level AMU indicators by U.S. dairy farms.

## 1 Introduction

Antimicrobial resistance (AMR) is a serious One Health concern threatening not just human, animal, and environmental health but also agricultural production and the economy (WHO, 2021). In 2019 alone, the global human health burden associated with bacterial AMR was an estimated 1.27 million deaths (Murray et al., 2022). By 2050, approximately ten million people could die from AMR annually (O’Neill, 2016). The mechanism of AMR emergence and spread is complex, but antimicrobial use (AMU) in food producing animals, dairy cattle included, is considered to contribute to the One Health burden associated with AMR (Marshall and Levy, 2011; Hoelzer et al., 2017; Ma et al., 2021).

The U.S. is one of the top countries in the world with respect to the size of the national dairy cattle population (FAOSTAT). According to the FDA, in the U.S. in 2020, medically important antimicrobials for use in cattle (beef and dairy cattle combined because data were not available for these two different production categories separately) accounted for 41% of the total sales of antimicrobials for use in food animals (FDA, 2021). On dairy farms, antimicrobials are used to treat bacterial infections, such as mastitis in lactating cows and respiratory disease in calves (Llanos-Soto et al., 2021; Casseri et al., 2022). Studies have suggested variable levels of association between the level of AMU on dairy farms and the emergence of AMR in the commensals and pathogens of dairy cattle (Snow et al., 2012; Duse et al., 2015; Gonggrijp et al., 2016; Hordijk et al., 2019). However, conclusive evidence that AMU in dairy farms leads to AMR infections that cause extended illnesses or deaths in dairy cattle is still lacking, implying the presence of multiple factors influencing the epidemiology of AMR diseases (de Verdier et al., 2012; Cummings et al., 2013; Owen et al., 2017; Bokma et al., 2020), as well as exposing the lack of quantitative data to allow causal inference (Cummings et al., 2013; Owen et al., 2017). Therefore, gathering quantitative data about AMU is a crucial step to understanding the relationship between AMU and the development of AMR (MacFadden et al., 2016) and informing antimicrobial stewardship (Redding et al., 2019; Schrag et al., 2020c; Cheng et al., 2022; Fonseca et al., 2022).

Scientists and governments worldwide have proposed different indicators to quantify AMU in cattle (Redding et al., 2019; Brault et al., 2019a; Schrag et al., 2020a; Cheng et al., 2022; Fonseca et al., 2022). An indicator is usually calculated using a division equation with different combinations of animal, antimicrobial, and temporal information as the numerator and denominator. Consequently, each indicator has a different focus, granularity, interpretation, and data requirements (Brault et al., 2019a; Schrag et al., 2020b). For example, an indicator that uses the mass of the active substance administered as the numerator and the population of animals at risk as the denominator (mg/100 cattle-at-risk) is easy to calculate but may be misleading because it does not consider the animal body mass and antimicrobial potency and dosage differences (Brault et al., 2019a). For mass-based indicators, the European Surveillance of Veterinary Antimicrobial Consumption (ESVAC) defined the population correction unit (PCU) to adjust antimicrobial sales data, where PCU is the product of the number of animals in the population and animal body mass at treatment (European Medicines Agency, 2019). The U.S. equivalent of the PCU denominator is the target animal biomass (TAB) developed by FDA, which also adjusts antimicrobial sales data (FDA Center for Veterinary Medicine).

Antimicrobial use can be quantified by several dose-based indicators as well, which are calculated by using different dose definitions and were developed by various research and government groups. Timmerman et al. adopted the used daily dose (UDD), which means the administered dose per day per kilogram of animal body mass (Timmerman et al., 2006). Jensen et al. defined the animal daily dose (ADD), which means the average maintenance dose for treatment in a specific species (Jensen et al., 2004). To harmonize and better monitor antimicrobial sales data in EU countries, ESVAC developed the defined daily dose (DDDvet) and the defined course dose (DCDvet) for animals, which provide standard parameters to adjust AMU data for different antimicrobials and animal species (European Medicines Agency, 2015). In addition, Schrag et al. defined the concepts of the standard regimen (SReg), which means the use of an antimicrobial product for a disease event in an animal and implicitly accounts for dose, therapy length, and the number of administrations (Schrag et al., 2020a; Schrag et al., 2020b). Schrag et al. used the counts and grouping of SRegs to quantify AMU (Schrag et al., 2020b; Schrag et al., 2022). While indicators that quantify AMU in terms of the applied course doses and regimens differ, the two terms, ‘course’ and ‘regimen’, refer to the same concept (defined above for regimen).

Countries like the Netherlands and Denmark have implemented national AMU surveillance systems that quantify AMU based on their national DDDvet (Kasabova et al., 2019; Moura et al., 2022). However, there is still a need for a national unified or recommended indicator(s) to quantify AMU on U.S. dairy farms. Many farms in the U.S. have yet to use different indicators to evaluate AMU, contributing to a poor understanding of AMU and its role in the emergence of AMR and missing the opportunity to inform antimicrobial stewardship (de Campos et al., 2021). Also, a review from 2022 reported that many U.S. dairy producers rely on their experience to make treatment decisions without veterinary input (Ruegg, 2022). Due to the inconsistent definitions of indicators for quantifying on-farm AMU, veterinarians and farmers need more guidance in selecting suitable indicators for quantifying and adjusting their AMU (Gozdzielewska et al., 2020; Moore et al., 2021; Ruegg, 2022). Quantification of AMU contributes to reducing costs of excess antimicrobials while keeping healthy dairy cattle, which is the primary motivation for dairy farmers to adjust AMU (Gozdzielewska et al., 2020). In addition, farmers are also interested in knowing how their AMU compares to other farms (Casseri et al., 2022). However, comparing their AMU with other farms requires sharing AMU data, which may raise privacy concerns. At the national level, detailed and accurate on-farm AMU data are the cornerstone of a national AMU monitoring system and provide support for developing interventions (Sanders et al., 2020).

The objectives of this study were to: (i) standardize published indicators for monitoring farm-level AMU in dairy cattle by standardizing their underlying variables and parameters and (ii) compare AMU indicators based on their data needs and effort, level of privacy concerns, and ability to capture trends and inform antimicrobial stewardship on the U.S. dairy farms. This information will provide guidelines for a more intuitive comparison and selection of AMU indicators by farmers and veterinarians, which can drive meaningful antimicrobial stewardship decisions on dairy farms and help evaluate the relationship between AMU and AMR.

## 2 Method

### 2.1 Indicator selection

We conducted a literature review to identify existing indicators that can quantify AMU on U.S. dairy farms. A total of 16 indicators were selected, and we categorized them into three groups: count-based (five), mass-based (two), and dose-based (nine).

The selected count-based indicators were all from Schrag et al.’s studies, which were the number of therapeutic events (*nTE*), number of standard regimens (*nREG*), antimicrobial regimen to therapy ratio (*RT-ratio*), number of regimen time frame days (*nRTFD*), and total length of all therapies (*nDOT*) (Schrag et al., 2020b; Schrag et al., 2022). These five indicators contain neither the total mass of antimicrobial administered nor the dose information in the calculation. Instead, the numerators are the number of therapeutic events, regimens, or days. The denominators for all five indicators are either the number of animals (*nTE*, *nREG*, *nRFTD*, and *nDOT*) or the number of therapeutic events (*RT-ratio*). Some of the information contained in the count-based indicators overlaps with the information contained in the dose-based indicators, but they are not identical. Because the count-based indicators don’t depend on the availability of globally accepted standard dose-related parameters needed for calculating the administrated dose (e.g., the defined daily dose (DDD)), they are simpler to calculate and interpret. Also, they are more robust since they are not affected by changes or variability in standard dose-related parameters over time and across farms.

The selected mass-based indicators were *mg/100 cattle-at-risk* (referred to as "*mg/100 animals-at-risk*" in our study) and *mg/TAB*. The *mg/100 cattle-at-risk* indicator is the easiest to calculate and interpret (Brault et al., 2019a). In this study, we used an adaptation of the FDA’s definition of *mg/TAB* for quantifying farm-level AMU that is otherwise applicable only to the national-level AMU. This was achieved by replacing in the calculation the national antimicrobial sales data with the farm-level AMU data and by using the farm-level specific animal body mass instead of the national standard animal body mass (FDA Center for Veterinary Medicine). We did not consider the EU indicator with the PCU denominator because the *mg/TAB* indicator has the same principle and is more suitable for the U.S. farming settings.

Most (nine) of the selected 16 indicators fall into the dose-based group. Specifically, we selected the number of study defined daily doses (*nDDDp*), the number of standard defined daily doses (*nDDDv*), the number of study defined course doses (*nDCDp*), and the number of standard defined course doses (*nDCDv*) from Schrag et al.’s study (Schrag et al., 2020b). Additionally, we selected indicators that combine the treatment frequency with the used daily dose (*TF_UDD_*) or standard defined daily dose (*TF_DDD_*) from Kasabova et al.’s study (Kasabova et al., 2019). Also, we selected the indicators from Brault et al.’s study quantifying the number of animal daily doses per 100 treated animals that use the individual animal AMU and body mass information (*nADD(kg_a_)/100 treated animals*) or use the average animal AMU and body mass information (*nADD(kg_m_)/100 treated animals*) (Brault et al., 2019a). Finally, we selected the number of Canadian-defined daily doses per 1,000 animal days at risk (*nDDDv/1,000 animal days-at-risk*) proposed by the Canadian Government (Canadian Integrated Program for Antimicrobial Resistance Surveillance).

### 2.2 Parameter standardization

The definitions of terms (variables and parameters) appearing in the equations for calculating the original AMU indicators are inconsistent. For example, the numerators in *TF_UDD_*, *ADD*-based indicators, and *nDCDp* describe the amount of antimicrobial used, and the unit in all three is mg of an active substance. However, the numerators in these three indicators are defined differently: as “the amount of active substance for every active compound” in *TF_UDD_*, “the quantity of active substance in mg administered” in *ADD*-based indicators, and “substance specific total milligrams” in *nDCDp* (Brault et al., 2019a; Kasabova et al., 2019; Schrag et al., 2020b). The subtle differences in definitions can cause confusion (Moore et al., 2021).

In addition, the indicators often use different methods to estimate animal body mass on a farm, and the body mass information often does not include the animal production category (e.g., unweaned calf, weaned calf, pregnant heifer, lactation #1). For example, Kasabova et al. estimated the animal body mass by rearranging the formula (equation (2) in Kasabova et al.) for calculation of the used daily dose (UDD), i.e., by dividing the mass of the administered active substance by the product of the number of treated animals, the recommended UDD, and treatment days (Kasabova et al., 2019); while Brault et al. used the mean animal body mass of animals on a feedlot at the time of exposure to any antimicrobial (Brault et al., 2019a); and Schrag et al. used the assumed animal body mass of 680 kg that is based on a prior study on the U.S. dairy farms (Schrag et al., 2020b).

To address these inconsistencies, we redefined variables and parameters based on the equations for each of the 16 selected indicators and expressed them in a standardized way. This included assigning identical definitions to the numerators with the same meaning and describing body mass variables/parameters in a way that the distinctions among them are obvious. We grouped all terms appearing in the indicator equations into: (i) data collected per treatment (C); (ii) composite records of collected data for each individual administrated treatment (*a*) or regimen (*R*) (CR); (iii) data collected periodically (e.g., weekly) (P); (iv) ‘farm standard’, a constant value obtained from a one-time calculation or approximation for a specific farm (FS); (v) ‘general standard’, a constant value available from the literature (GS); and (vi) the derived terms (D). The terms (i)-(v) represent the ‘primary data’ required for the calculation of indicators that need to be assembled by a farmer/veterinarian (**Table 1**), while the ‘derived terms’ in (vi) are calculated from the collected/identified primary data or other calculated terms and they are presented as an intermediate step for ease of indicator calculation and comparison (**Table 2**). Additionally, we categorized all terms (primary data and derived terms) into three categories based on the fundamental requirements for estimating an AMU indicator: antimicrobial, animal, and time. We have standardized definitions of terms while maintaining their original meaning so that the identical components in calculation can be easily identified across all indicators. This also achieved interchangeability between indicators. For example, the definition of the animal body mass now is the same for *mg/TAB*, *nDDDp*, *nDCDp*, *TF_UDD_*, and *nADD(kg_m_)/100 treated animals*, which is farm-specific average body mass for the production category of the treated animal. Therefore, users can use the same body mass data for these five indicators.

**Table 1:** Definition of primary data (variable and standard parameter terms) required for calculation of farm-level antimicrobial drug use indicators.

Category	Notation	Definition (unit)	Type^1^	Source
Animal	*i*	Individual animal identification number on a farm *f* for an animal that was treated with an antimicrobial product (animal)	C	NA^2^
	*d*	Specific treatment indication/disease syndrome (treatment indication)	C	Adapted from (Schrag et al., 2020a)
	*p*	Specific production category of a treated animal at the time of antimicrobial product administration (production category)	C	NA
	*n_wk,p_*	Number of animals of a given production category (*p*) present on a farm *f* in a given week (*wk*) (animal)	P	Adapted from (Schrag et al., 2020a)
	*w_i_*	Body mass of an individually treated animal at the time of antimicrobial product administration (can be measured or estimated from animal age at the time of treatment using growth charts) (kg)	C	Adapted from (Kasabova et al., 2019)
	*w_f,p_*	Farm *f* specific average body mass (or farm-specific standard body mass) for the production category *p* of a treated animal at the time of antimicrobial product administration. Can be obtained from historical farm records or by measuring a representative subset of animals (kg)	FS	Adapted from (Brault et al., 2019a)
	*w_p_*	Standard average body mass for the production category *p* of a treated animal at the time of drug product administration (kg)^3^	GS	(Canadian Integrated Program for Antimicrobial Resistance Surveillance; European Medicines Agency, 2023)
Antimicrobial	*s*	Specific administrated active substance (*s*) (active substance)	C	NA
	*r*	Specific route of antimicrobial product administration (administration route)	C	NA
	$m_{s}$	Mass of an active substance (*s*) in a single administration of an antimicrobial product (listed on the product label) (mg)	GS	NA
	$m_{s_{i}}$	Mass of an active substance (*s*) actually administrated in a single administration of an antimicrobial product, including for extra-label use. Recorded only if different from the mass (*m_s_*) listed on the product label (mg)	C	NA
	$C_{R}$	Prescribed number of antimicrobial product administrations as part of a single regimen (administration)	GS/FS	NA
	$c_{R_{i}}$	The actual number of antimicrobial product administrations as part of a regimen administrated to animal *i*. Recorded only if different from the general/farm standard (*c_R_*) for the regimen (administration)	C	NA
	*AD_i_*	The actual dose (*m_i_*/*w_i_*) of an active substance (*s*) in a single antimicrobial administration for a therapeutic purpose targeting a single disease event (*d*) in an individual animal (*i*) (mg active substance/kg animal)	C	Adapted from (Brault et al., 2019a; Schrag et al., 2020a)
	*AD_m_*	Prescribed or mean dose of an active substance (*s*) in a single antimicrobial administration for a therapeutic purpose targeting a single disease event (*d*) in an individual animal (*i*) (mg active substance/kg animal)	GS/FS	Adapted from (Brault et al., 2019a; Schrag et al., 2020a)
	*DDDv*	Standard defined daily dose by the European Surveillance of Veterinary Antimicrobial Consumption or Government of Canada (mg active substance/kg animal/day)^4^	GS	(Canadian Integrated Program for Antimicrobial Resistance Surveillance; Defined daily doses for animals (DDDvet) and defined course doses for animals (DCDvet))
	*DCDv*	Standard defined course dose proposed by European Surveillance of Veterinary Antimicrobial Consumption or Government of Canada (mg active substance/kg animal/course)^4^	GS	(Canadian Integrated Program for Antimicrobial Resistance Surveillance; Defined daily doses for animals (DDDvet) and defined course doses for animals (DCDvet))
	*a*	Single administration: Antimicrobial product administered at a single restraining event to an individual animal (*i*). Dataset associated with each individual administration: a={i, t, r, s, m, d, p, w} (administration)	CR	Adapted from (Schrag et al., 2020a)
	*R*	Standard regimen (course): Recorded antimicrobial product administration(s) for a therapeutic purpose targeting a single disease event (*d*) in an individual animal (*i*). Multiple administrations in an animal (*a_i_*) are counted as part of a single regimen when product administrations are consecutive, never resulting in a time gap between administrations of greater than the pre-determined administration interval of 5 days. Dataset associated with each individual administrated regimen: $R=\left\{i,\;t_{first},\;t_{last},\;r,\;s,\;m,\;d,\;p,\;\mathrm{w,}\;c_{R},\;int,\;adjF\right\}$ (regimen)	CR	Adapted from (Schrag et al., 2020b)
Time	*t*	The date of an individual single administration of an antimicrobial product to an individual animal (*i*) at a single restraining event. In the case of a regimen, *t_first _* and *t_last _* denote the first and last day of the regimen (date)	C	NA
	*int*	Interval between administrations within a single regimen that is less than 24h (day)	GS	(Schrag et al., 2020b)
	*adjF*	Adjustment factor for long-acting antimicrobial products, for which single administration provides > 1 day of therapy. Can be the time interval between administrations or the estimated duration of antimicrobial effect (unitless)	GS	Adapted from (Schrag et al., 2020b)
	*ADR*	Average days at risk: an average number of days individual animals of production category *p* are present on farm *f* (days)^5^	GS/FS	Adapted from (Canadian Integrated Program for Antimicrobial Resistance Surveillance)

^1^Term types: C, collected per treatment; P, collected periodically (e.g., weekly); FS, farm standard (obtained from a one-time calculation or approximation for a specific farm); GS, general standard (available from the literature); CR, composite data for each individual administrated treatment (a) or regimen (R).

^2^NA, not applicable.

^3^Examples of general standard body mass values available from the literature: From Schrag et al.’s paper, lactating cow w_p_=680kg (Schrag et al., 2020b); From the FDA: livestock dairy cows w_p_=635.03 kg (FDA, 2022); From the European ESVAC standard: Veal calves w_p_=80kg, dairy cattle w_p_=500kg, meat cattle w_p_=500kg (European Medicines Agency, 2023).

^4^Currently defined for pigs, cattle and poultry. For example, DDDv and DCDv for oral route Amoxicillin in cattle are 20 mg/kg and 81 mg/kg, respectively; DDDv and DCDv for oral route Ampicillin in cattle are 29 mg/kg and 123 mg/kg, respectively (Defined daily doses for animals (DDDvet) and defined course doses for animals (DCDvet)).

^5^Examples of the general standard average length of stay in a production category available from the literature: e.g., unweaned calves= 2 months; heifers= 13 months (Lang, 2017).

**Table 2:** Definition of derived terms required for calculation of farm-level antimicrobial drug use indicators^1^.

Category	Notation	Definition (unit)	Equation	Source
Animal	*D*	List of all treatment indications (diseases; *d*) treated with antimicrobial products on a farm *f* during a period of time *T* (categorical)^3^	D={digestive, mastitis,…}	NA^2^
	*P*	List of all animal production categories (*p*) present on a farm *f* during a period of time *T* (categorical)^3^	P={calf, parity 1, ...}	NA
	*n_wk_*	Number of animals of any production category present on a farm *f* in a given week (*wk*) (animal)	$\sum_{p\in P}n_{wk,p}$	Adapted from (Schrag et al., 2020a)
	$\bar{N_{p}}$	Average number of animals of a given production category (*p*) on a farm *f* (or average farm inventory of a given production category (*p*)) during a time period *T* (animal)	$\frac{\sum_{wk=1}^{tw}n_{wk,p}}{tw}$ , where tw=floor(T/7)	Adapted from (Schrag et al., 2020a)
	*K*	Total number of animals on a farm *f ever* treated with an antimicrobial product during a time period *T.* Can be calculated overall (*K*), or subset for a specific production category (*p*), active substance ( s ), route of administration ( r ), disease (*d*), or their combination (animal)	$\#\left(i_{R\;\geq 1}\right)|t\in \left\{1,..T\right\}$	NA
Antimicrobial	*S*	List of all active substances (*s*) administered on a farm *f* during a period of time *T* (categorical)^3^	S={Tetracycline, Sulfonamide, …}	NA
	*RA*	List of routes of antimicrobial product administration (categorical)^3^	RA={intramuscular, subcutaneous, …}	NA
	$a_{T}$	Total number of all single antimicrobial administrations (a) administered on a farm *f* during a period of time *T.* Can be calculated overall ( $a_{T}$ ), or subset for a specific production category (*p*), active substance ( s ), route of administration ( r ), disease (*d*), or their combination (administration)	#(a) |t∈{1,..T}	
	*R_T_*	Total number of all standard regimens (*R*) administered on a farm *f* during a period of time *T*. Can be calculated overall ( $R_{T}$ ), or subset for a specific production category (*p*), active substance (*s*), route of administration (*r*), disease (*d*), or their combination. (regimen)	$\#\left(R_{d}\right)|t\in \left\{1,..T\right\}$	Adapted from (Schrag et al., 2020b)
	$m_{R}$	Total mass of an active substance (*s*) over all administrations (*c*) administrated as part of a specific single regimen in an individual animal (*i*) (mg)	$c_{R}m_{s}$ or $\sum_{a_{i}=1}^{c_{R_{i}}}m_{s_{i}}$	Adapted from (Schrag et al., 2020a)
	$\bar{m_{R}}$	Mean mass of an active substance (*s*) over all instances of application of a specific regimen administrated during a period of time *T* ( $m_{R}$ ) (mg)	$\frac{\sum_{R_{s,r}=1}^{R_{T}^{s,r}}m_{R_{s,r}}}{R_{T}}$	NA
	*m_p,s_*	Total mass of an active substance (*s*) used in an animal production category (*p*) on farm *f* during a period of time *T* (mg)	$\sum_{a_{p,s}=1}^{a_{T_{p,s}}}m_{a_{p,s}}$	NA
	*m_p_*	Total mass of all active substances used in an animal production category (*p*) on farm *f* during a period of time *T* (mg)	$\sum_{s\in S}m_{p,s}$	NA
	*ADD_i_*	Actual daily dose for an active substance (*s*) in a single antimicrobial administration for a therapeutic purpose targeting a single disease event (*d*) in an individual animal (*i*) (mg active substance/kg animal/day)	$\frac{AD_{i}\;}{adjF}$ or $\frac{AD_{i}\;}{int}$	Adapted from (Brault et al., 2019a)
	*ADD_m_*	Prescribed or mean daily dose for an active substance (*s*) in a single antimicrobial administration for a therapeutic purpose targeting a single disease event (*d*) in an individual animal (*i*) (mg active substance/kg animal/day)	$\frac{AD_{m}\;}{adjF}$ or $\frac{AD_{m}\;}{int}$	Adapted from (Brault et al., 2019a)
	*UDD*	Median (preferred) or mean^4^ of actual used daily doses administered per day as part of a regimen per actual kg of animal body mass at the time of treatment (*w_R_*) on farm *f* during a time period *T* (mg active substance/kg animal/day)	median ( $\frac{m_{R}}{w_{R}\times \text{DOT}}$ ) or $\frac{\sum_{R=1}^{R_{T}}\frac{m_{R}}{w_{R}\times \text{DOT}},}{R_{T}},$ where $w_{R}=w_{i}$ at tfirst of R	Adapted from (Kasabova et al., 2019)
	*DDDp*	Study-defined daily dose that is specific for the population under study (mg active substance/kg animal/day)	$\frac{\bar{m_{R}}\;}{\text{DOT}}\times \;\frac{1}{w_{f,p}}$	Adapted from (Schrag et al., 2020b)
	*DCDp*	Study-defined course dose that is specific for the population under study (mg active substance/kg animal/course)	$\frac{\bar{m_{R}}\;}{w_{f,p}}$	Adapted from (Schrag et al., 2020b)
Time	*DOT*	Duration of treatment. Depending on antimicrobial product used, *DOT* is expressed as: *cDOT*: Count of calendar days on which treatment was administered as part of a single regimen, used for antimicrobials administered in intervals ≤1 day; *aDOT*: Adjusted length of therapy for a single regimen used for a long-acting antimicrobial product or product administered in intervals > 1 day. (day)	DOT = {cDOT, aDOT} $cDOT=\{\begin{array}{c} c\times int,\;int<1\;day\\ c,\;int=1\;day \end{array}$ aDOT=c×adjF	Adapted (Schrag et al., 2020b)
	*TE*	Total number of therapeutic events among treated animals. Each therapeutic event is identified by grouping regimens in an individual animal by date of administration so that regimens within 7 days are part of the same treatment event (event)	NA	(Schrag et al., 2022)
	*cfl_R_*	The number of calendar days between the first and last administration of a regimen to an animal (*i*) (day)	$t_{last}R-t_{first}R$	Adapted from (Schrag et al., 2020a)

^1^Data collected for calculation of antimicrobial use indicators on a farm f are defined in **Table 1**. The time period T of data recording for periodic calculation of an antimicrobial use indicator is user defined (e.g., month, quarter, year).

^2^NA, not applicable.

^3^Examples for levels of categorical variables listed in the set are for illustration purposes.

^4^Median is preferable when the distribution of applied UDDs is skewed, however, the mean is acceptable and is easier to calculate on a farm.

For all AMU indicators, the time period *T* of data recording for periodic calculation of an AMU indicator is user defined (e.g., month, quarter, year). We summarized the definitions, notations, and sources of primary data and derived terms used in calculating 16 AMU indicators in **Tables 1**, **2**. In **Table 1**, we included specific terms as subscripts to describe AMU: treatment indication, production category, active substance, and route of administration. These terms are crucial for accurate calculations and meaningful comparisons and interpretation of indicators, and will be helpful in evaluating implications of AMU (e.g., when comparing intramammary vs parenteral therapies with the count-, mass- and dose-based indicators and evaluating implications of these therapies for AMR). We showed how these terms are used in calculating AMU indicators in **Table 3**. For transparency, the new and original definitions of the terms are shown side-by-side in **Supplementary Table S1**. Definitions of main abbreviations and acronyms used in derivation and evaluation of antimicrobial use indicators are provided in alphabetical order in **Supplementary Table S2**. We also created a simplified dataset for a hypothetical dairy farm, and we illustrated step by step how to obtain all values listed in **Tables 1**–**3**, which respectively include primary data, derived terms, and the indicators for quantifying AMU on the farm (**Supplementary Data**).

**Table 3:** Formulas for calculation of antimicrobial drug use indicators^1^.

Group	Indicator	Definition	Equation^2^	Reference
Count-based	*nTE*	Number of therapeutic events per animal of a given production category (*p*) on farm *f* during a time period *T*. (therapeutic events/animal)	$\frac{TE}{\bar{N_{p}}}$	Adapted from (Schrag et al., 2022)
	nREG	Number of regimens per animal of a given production category (*p*) on farm *f* during a time period *T*. (regimens/animal)	$\frac{R_{T}}{\bar{N_{p}}}$	Adapted from (Schrag et al., 2020b)
	*RT-ratio*	Antimicrobial regimen to therapy ratio (RT-ratio), calculated by dividing the number of antimicrobial regimens by the number of therapeutic events. (regimens/therapeutic event)	$\frac{nREG}{nTE}$	(Schrag et al., 2022)
	*nRTFD*	Regimen time frame days (RTFD) per animal of a given production category (*p*) on farm *f* during a time period *T*. Numerator is estimated as the sum of *cfl_R_* (days/animal)	$\frac{\sum_{R=1}^{R_{T}}cfl_{R}}{\bar{N_{p}}}$	Adapted from (Schrag et al., 2020b)
	*nDOT*	Total length of all therapies in days per animal of a given production category (*p*) on farm *f* during a time period *T.* (days/animal)	$\frac{R_{T}\;\times \;DOT}{\bar{N_{p}}}$	Adapted from (Schrag et al., 2020b)
Mass-based	*mg/TAB*	Total mass of all active substances used per animal biomass of a given production category (*p*) treated with these active substances on farm *f* during a time period *T.* (mg active substance/kg animal)	$\frac{m_{p}}{w_{f,p}\times \bar{N_{p}}}$	Adapted from (European Medicines Agency, 2023; FDA Center for Veterinary Medicine)
	*mg/100 animals-at-risk*	Total mass of all active substances used per 100 animals-at-risk of a given production category (*p*) on farm *f* during a time period *T.* (mg active substance/animal)	$\frac{m_{p}}{\bar{N_{p}}}\times 100$	(Brault et al., 2019a)
Dose-based	*nDDDp*	Number of study-defined daily doses per animal of a given production category (*p*) for the farm *f* during a time period *T.* (doses/animal)	$\frac{\frac{m_{p,s}}{DDDp\times \;w_{f,p}}}{\bar{N_{p}}}$	Adapted from (Schrag et al., 2020b)
	*nDDDv*	Number of the standard defined daily doses per animal of a given production category (*p*) on farm *f* during a time period *T*. (doses/animal)^3^	$\frac{\frac{m_{p,s}}{DDDv\times \;w_{p}}}{\bar{N_{p}}}$	(Schrag et al., 2020b)
	*TF_UDD_*	Treatment frequency per animal of a given production category (*p*) on farm *f* based on the median (preferred) or mean Used Daily Dose for a drug product with active substance *s* during a time period *T.* (doses/animal)	$\frac{m_{p,s}}{UDD\times w_{f,p}\times \bar{N_{p}}}$	(Kasabova et al., 2019)
	*TF_DDD_*	Treatment frequency per animal of a given production category (*p*) on farm *f* based on standard (EU) defined daily doses for a drug product with active substance *s* during a time period *T.* (doses/animal)^3^	$\frac{m_{p,s}}{DDDv\times w_{p}\times \bar{N_{p}}\;}$	(Kasabova et al., 2019)
	*nADD(kg_a_)/100 treated animals*	Number of actual individually administered daily doses per 100 treated animals of a given production category (*p*) on farm *f* during the time period *T*. Estimated by accounting for the actual administered dose and the actual body mass (kg) of treated animals. Can be interpreted as: how many days on average 100 animals on farm *f* were treated during a time period *T*. (doses/animal)	$\sum_{\text{i}=1}^{R_{T}}\frac{m_{s,i}}{w_{i}\times ADD_{i}}\times \frac{100}{K_{p}}$	(Brault et al., 2019a)
	*nADD(kg_m_)/100 treated animals*	Number of prescribed or individually administered mean daily doses per 100 treated animals of a given production category (*p*) on farm *f* during the time period *T.* Estimated by accounting for the standard administered dose and the mean body mass (kg) of treated animals. Can be interpreted as: how many days on average 100 animals on the farm *f* were treated during a time period *T*. (doses/animal)	$\frac{m_{p,s}}{w_{f,p}\times ADD_{m}}\times \frac{100}{K_{p}}$	Adapted from (Brault et al., 2019a)
	*nDDDv/1,000 animal days-at-risk*	Number of Canadian-defined daily dose per 1,000 animal-days-at-risk of a given production category (*p*) on farm *f* during a time period *T.* (doses/animal-days-at-risk)	$\frac{m_{p,s}/DDDv}{ADR\times w_{p}\times \bar{N_{p}}}\times 1000$	(Canadian Integrated Program for Antimicrobial Resistance Surveillance)
	*nDCDp*	Number of study-defined course doses per animal of a given production category (*p*) for the farm *f* during a time period *T.* (courses/animal)	$\frac{\frac{m_{p,s}}{DCDp\times \;w_{f,p}}}{\bar{N_{p}}}$	Adapted from (Schrag et al., 2020b)
	*nDCDv*	Number of standard defined course doses per animal of a given production category (*p*) on farm *f* during a time period *T*. (courses/animal)	$\frac{\frac{m_{p,s}}{DCDv\times \;w_{p}}}{\bar{N_{p}}}$	(Schrag et al., 2020b)

^1^Equations are illustrated for estimating indicators for a single active substance except for *mg/TAB* and *mg/100 animals-at-risk*, which by definition represent the use of all administrated active substances. Additionally, equations illustrate the estimation of indicators for a given animal production category.

^2^Terms in the equations are defined in **Tables 1**, **2**.

^3^If *nDDDv* and *TF_DDD_* use the same DDDv (Standard defined daily dose by the European Surveillance of Veterinary Antimicrobial Consumption or Government of Canada (mg active substance/kg animal/day)), they will result in identical values of nDDDv and TF_DDD_ indicators.

### 2.3 Indicators’ comparison

To assess the data needs and interchangeability of indicators, we cross-tabulated the 16 indicators and the data/terms needed for their calculation. Additionally, to compare the 16 indicators, ZL, EB, and RI established six data requirement- and five stewardship information-driven criteria for evaluation (**Table 4**). The data requirements focused on data needs and evaluated the level of detail provided by an indicator about (i) the actual animals treated (Animal information, ani); (ii) antimicrobials used (Exposure data, ed); (iii) ability to detect extra-label use (Extra-label use, el); (iv) the ease of implementation (Ease of data recording and calculation, edr); (v) standard parameter use (Standard parameters, sp); and (vi) the potential for privacy concerns regarding sharing of indicators or data used for their calculation (Privacy concerns, pc). Among these data requirements, the criteria ani, ed, and el relate to the accuracy of the AMU measurement, which we call accuracy criteria. Here, the term accuracy is defined in terms of granularity and exactness. Granularity means the level of actual and detailed information an indicator will include, and exactness is used to represent the absence of standard parameters (FS or GS) in the indicator calculation. Therefore, in this study, we evaluate the indicators’ procedural accuracy, which describes their capacity or potential to capture the true application of an antimicrobial, rather than their field accuracy when these indicators are applied in a farm setting. The procedural accuracy is necessary but not sufficient to achieve the field accuracy as an indicator with great granularity and exactness may be inaccurate in a field application, for example, because of incorrectly recorded or missing data. The involved effort and privacy were represented by edr and pc criteria, respectively. The stewardship information criteria assessed the type of inference available from an indicator to inform AMU stewardship. These criteria evaluated whether an indicator can (i) monitor AMU in specific animal groups (Trends over time regarding treated animals, tt); (ii) track changes in the population at risk (Trends over time regarding population at risk, tp); (iii) track changes in the proportion of sick animals treated (Trends over time regarding treatment effort, tte); and track changes in the antimicrobial exposure in terms of (iv) antimicrobial substance (texam) and (v) length of treatments (texle). The characteristics and relationship of terms in the formula for calculation of the indicators were the most important factors for the score. ZL and EB independently scored the indicators with scores 1 (worst) – 5 (best) for each of the 11 criteria and discussed any differences in scores with RI until a consensus was reached. We considered the 5-point scale to be sufficient to distinguish the performance of each indicator in each criterion. **Supplementary Tables S3, S4** provide a detailed rationale for each score for the data- and stewardship-driven criteria, respectively. To interpret scores, we compared indicator scores with respect to (i) accuracy (ani, ed, and el), (ii) effort (edr), (iii) privacy (pc), and (iv) stewardship (tt, tp, tte, texam, and texle overall and individually). Due to the absence of established weights that different criteria should be given in comparisons, all criteria were given the same weight, and multiple criteria for accuracy and stewardship were averaged. The averaging process for accuracy and stewardship criteria ensured that the four criteria groups ((i)-(iv)) had the same scale, which was necessary to allow their direct and fair comparison. To aid interpretation, indicator scores for the four criteria groups were visually evaluated using a spider plot.

**Table 4:** Definition of criteria used for scoring antimicrobial drug use indicators.

Group	Criteria (abbreviation)	Definition
Data requirement	Animal Information (ani)	Use of the actual number of treated or total animals and the actual animal body mass for an individual animal in an indicator calculation
	Exposure data (ed)	Use of the actual amount (mass or dose) of antimicrobial administrated in the treatment of an individual animal in an indicator calculation
	Extra-label use (el)	Ability to account for the extra label use (i.e., antimicrobial use per kg animal, treatment interval, or treatment protocol that is not in accordance with the approved labeling)
	Standard parameters (sp)	Use of standard parameters for animal body mass and/or dose
	Privacy concerns (pc)	The level of privacy concerns associated with sharing data used for the calculation of the indicator or sharing the indicator value itself
	Ease of data recording and calculation (edr)	The ease of recording or obtaining data for calculation of an indicator and/or the complexity of involved calculations
Stewardship information	Trends over time regarding treated animals (tt)	Provides information about changes in specific groups of animals receiving an antimicrobial treatment (in terms of individual characteristics (body mass (*w*), production category (*p*), treatment indication (*d*))
	Trends over time regarding population at risk (tp)	Accounts for changes in the population at risk of antimicrobial treatment in a herd (through $\bar{N_{p}}$ ) or number of treated animals (through *Kp*)
	Trends over time regarding treatment effort (tte)	Provides information about the proportion of diseased animals in a herd that are receiving treatment
	Trends over time regarding exposure: antimicrobial substance (texam)	Provides information about changes in exposure to a specific antimicrobial substance (in terms of the amount (mass or dose) of the antimicrobial substance used in a herd or production category).
	Trends over time regarding exposure: length (texle)	Provides information about changes in the total length of antimicrobial treatments (through *DOT*)

## 3 Results

### 3.1 Required parameters and standardization

In the standardization of terms used in the calculation of AMU indicators, we focused on the mass of active substances, animal population, animal body mass, and treatment days (**Tables 1**, **2**) because these terms are the essential components of most indicators (**Table 3**). The standardization of active substance mass, which always appears in the numerator of the AMU indicator, resulted in four types of this parameter that are directly used for the calculation of the indicators: the total mass of an active substance administered for an individual animal in one regimen (*m_R_*), the mean mass of an active substance over all regimens ( $\bar{m_{R}}$ ) recorded during a defined period of time *T*, the total mass of an active substance used in an animal production category (*m_p,s_*) during a defined period of time *T*, and the total mass of all active substances used in an animal production category (*m_p_*) over a defined period of time *T*. Parameters *m_R_*, $\bar{m_{R}}$ , and *m_p,s_* are calculated separately for each active substance used on a farm. This allows tracking the use of individual drug classes and calculation of AMU indicators for individual active substances. The only exceptions are the mass-based indicators which use *m_p_* and thus calculate the total mass of all active substances combined, masking differences in the AMU across drug classes and the related indications for their use. Two kinds of the animal population parameters appeared in the denominators of indicators: the average number of animals in a production category ( $\bar{N_{p}}$ ) and the total number of treated animals in a production category (*K_p_*). The two *ADD*-based indicators use the *K_p_* parameter, while all other indicators except *RT-ratio* use $\bar{N_{p}}$ (**Table 3**).

Standardization of animal body mass resulted in three types of data: the measured or estimated body mass of an individual treated animal (*w_i_*), the average body mass for animals of the treated animal production category on a specific farm (*w_f,p_*), and standard average body mass for the treated animal production category (*w_p_*). We adapted the definition of duration of treatment (*DOT*) from Schrag et al.’s study to standardize the treatment time information in the indicators (Schrag et al., 2020b). For drugs with administration intervals ≤ 24 hours, the *DOT* is the count of calendar days of treatment (**Table 2**). For drugs with administration intervals greater than 24 hours, the *DOT* is the multiplication of the number of administrations and the time interval between administrations or the estimated duration of effect (**Table 2**).

A cross-tabulation of AMU indicators and their standardized terms showed that *TF_UDD_* required the most variables/parameters for calculation while *nREG* and *mg/100 animals-at-risk* required the least (**Table 5**). Dose-based indicators tended to require more variables/parameters than count-based and mass-based indicators because they included dose and additional animal information. Some of the indicators provide flexibility regarding the required data accuracy (specifically regarding terms describing animal body mass). In **Table 5**, we show this by using superscripts “*a*” and ‘*b*” when there are two options for a variable/parameter, with the superscript “*a*” indicating the preferred, more accurate option but which also requires more detailed data. For indicators *mg/TAB*, *nDDDp*, *TF_UDD_*, *nADD(kg_m_)/100 treated animals*, and *nDCDp*, the preferred option is *w_f,p_* because it represents the farm-specific average animal body mass. However, users who prefer a less time-consuming option for estimating *w_f,p_*, have privacy concerns regarding the animal body mass records or do not have the data available can use one of the available general standards, such as the FDA-estimated standard body mass of 635.03kg for dairy cows (FDA, 2022). When calculating *ADD*-based indicators and *TF_UDD_*, the preferred option for the numerator is *m_R_*, but $\bar{m_{R}}$ is also acceptable.

**Table 5:** Cross-tabulation of the required collected primary data and derived terms (variables and standard parameters) for estimating antimicrobial drug use indicators^1^ .

Indicator	Antimicrobial: Primary data (Derived term)									Animal: Primary data (Derived term)					Time: Primary data (Derived term)		
	*m,c_R_* (*m_R_*)	*m,c_R_,R* $\left(\bar{m_{R}}\right)$	*m,p,s* (*m_p,s_*)	*m,p* (*m_p_*)	*R* (*R_T_*)	*m,w* (*AD_i_*)	*AD_m_*	*DDDv*	*DCDv*	$n_{wk,p}$ $(\bar{N_{p}}$ )	*i_R_* (*K_p_*)	*w_i_*	*w_f,p_*	*w_p_*	*t* (*cfl_R_*)	*c,int/adjF* (*DOT*)	*ADR*
*nTE*					×					×					×		
*nREG*					×					×							
*RT-ratio*					×										×		
*nRTFD*					×					×					×		
*nDOT*					×					×						×	
*mg/TAB*				×						×			$\times^{a}$	$\times^{b}$			
*mg/100 animals-at-risk*				×						×							
*nDDDp*		×	×							×			$\times^{a}$	$\times^{b}$		×	
*nDDDv*			×					×		×				×			
*TF_UDD_*	$\times^{a}$	$\times^{b}$	×		×					×		$\times^{2}$	$\times^{a}$	$\times^{b}$		×	
*TF_DDD_*			×					×		×				×			
*nADD(kga)/100 treated animals*	$\times^{a}$	$\times^{b}$			×	$\times^{a}$	$\times^{a}$				×	×				×	
*nADD(kgm)/100 treated animals*			×				×				×		$\times^{a}$	$\times^{b}$		×	
*nDDDv/1,000 animal days-at-risk*			×					×		×				×			×
*nDCDp*		×	×							×			$\times^{a}$	$\times^{b}$			
*nDCDv*			×						×	×				×			

^1^Superscript letters “a” and “b” are used when there are two choices for a variable in the calculation of an indicator, where “a” indicates the preferred more accurate choice (according to the indicator’s definition), and “b” indicates the acceptable alternative.

^2^When calculating the Used Daily Dose (UDD) explained in **Table 2**, w_i_ is the preferred choice but w_f,p_ is also acceptable.

The most used terms *m_p,s_* and $\bar{N_{p}}\;$ appeared in eight and thirteen indicators, respectively. Mass-based indicators use *m_p_* as the numerator because they do not account for the mass of individual active substances. Count-based indicators require *R_T_* (sum of all standard regimens over a defined period of time *T*) because they are based on standard regimens and need to account for all administrated regimens to quantify AMU. Only *ADD*-based indicators require individual dose information for calculation. The indicators *nDDDv*, *TF_DDD_*, *nDCDv*, and *nDDDv/1,000 animal days-at-risk* need ESVAC and Canada-defined standard daily dose and course dose, which can be replaced with the U.S.-specific standard doses when they become available. The number of calendar days between the first and last administration in a regimen (*cfl_R_*) is required for the calculation of *nRTFD*, knowing the start dates (*t*) of regimen treatments for individual animals is necessary for the calculation of *nTE* and, therefore, also for *RT-ratio*, while the average days at risk (*ADR*) is needed for *nDDDv/1,000 animal days-at-risk*.

In addition to providing a visual comparison of the required primary data and derived terms for each indicator, **Table 5** provides the basis for creating education materials to guide farmers and veterinarians in selecting suitable indicators based on the data they have available. For example, if a farm only records the total amount of an active substance used and only has an average animal body mass at the animal production level rather than at an individual animal level, *nADD(kg_m_)/100 treated animals* is an applicable indicator, but *TF_UDD_* and *nADD(kg_a_)/100 treated animals* cannot be calculated. Two options of parameters in the same category, e.g., *w_f,p_* and *w_p_*, applicable to some indicators, add flexibility and simplicity. For example, if a farm does not have data to infer the farm-specific average animal body mass, they can use the FDA-estimated standard animal body mass to calculate *nADD(kg_m_)/100 treated* animals. Furthermore, a user can refer to **Table 5** to plan data collection based on the AMU indicator(s) they want to use in the future.

### 3.2 Scoring

**Figure 1:**
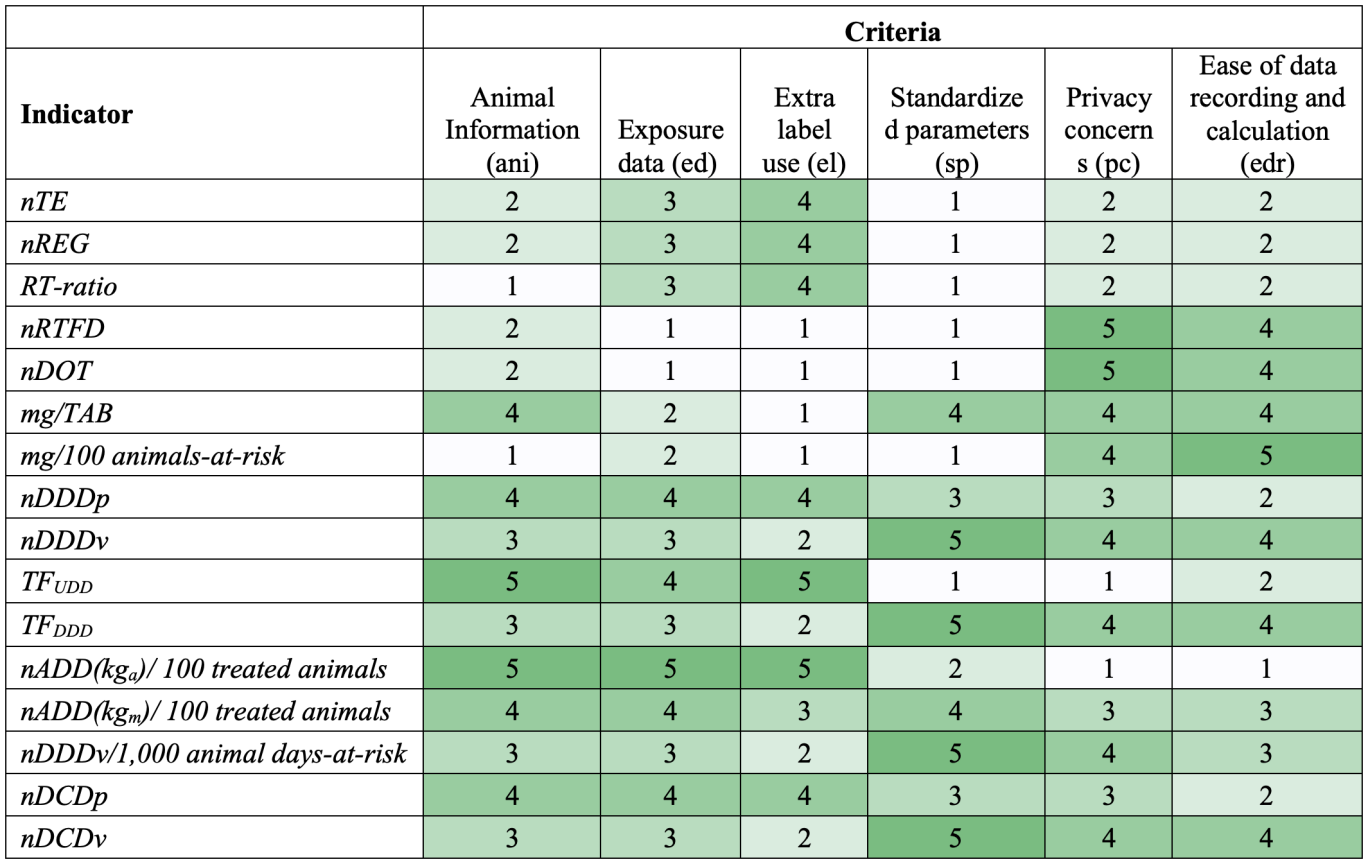
Heatmap showing antimicrobial drug use indicator scores based on the data requirement criteria (1=worst, 5=best). Colors range from white (worst) to dark green (best). Criteria are defined in **Table 4**, and each score is explained in **Supplementary Table S3**.

**Figure 2:**
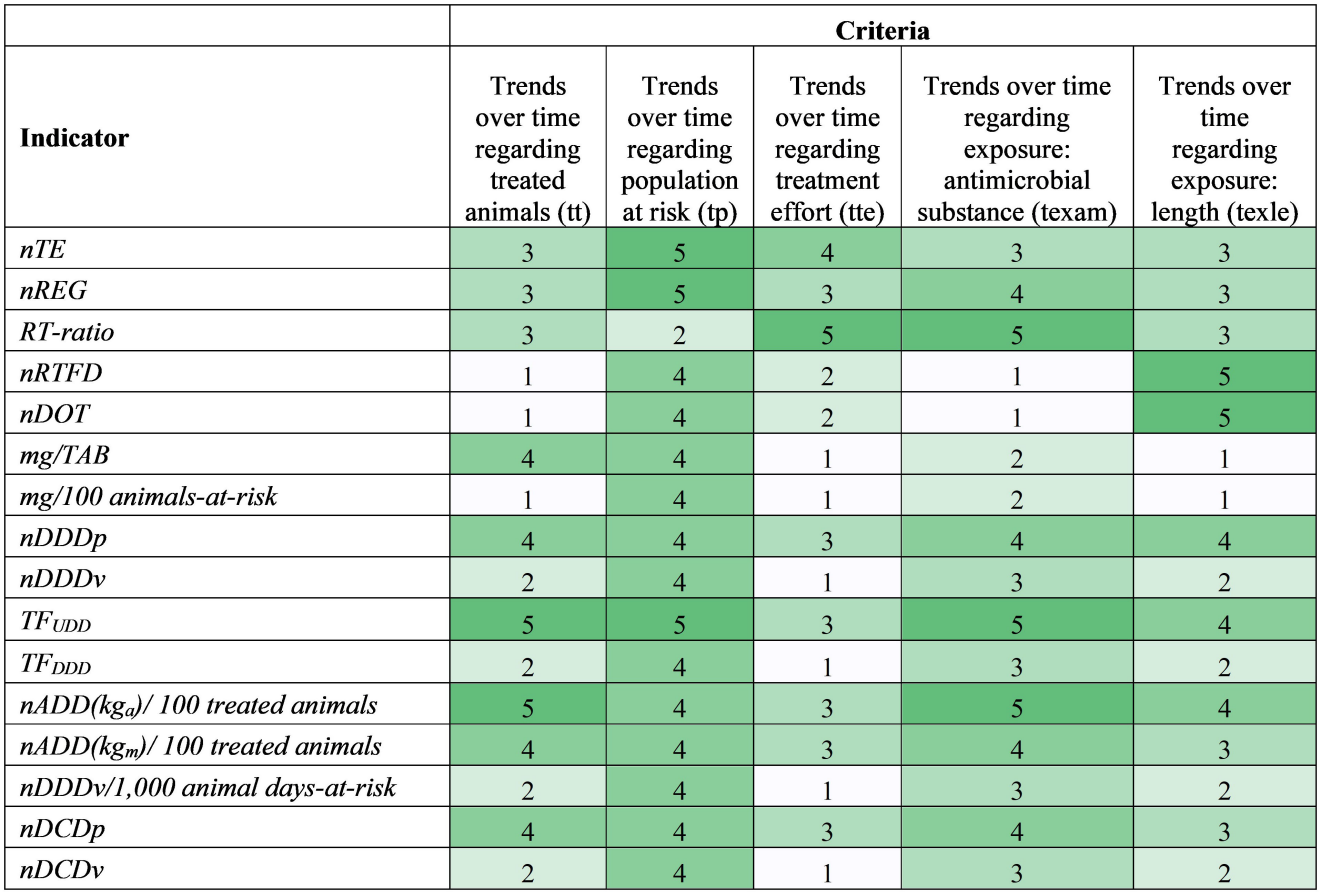
Heatmap showing antimicrobial drug use indicator scores based on the stewardship-driven criteria (1=worst, 5=best). Colors range from white (worst) to dark green (best). Criteria are defined in **Table 4**, and each score is explained in **Supplementary Table S4**.

**Figure 3:**
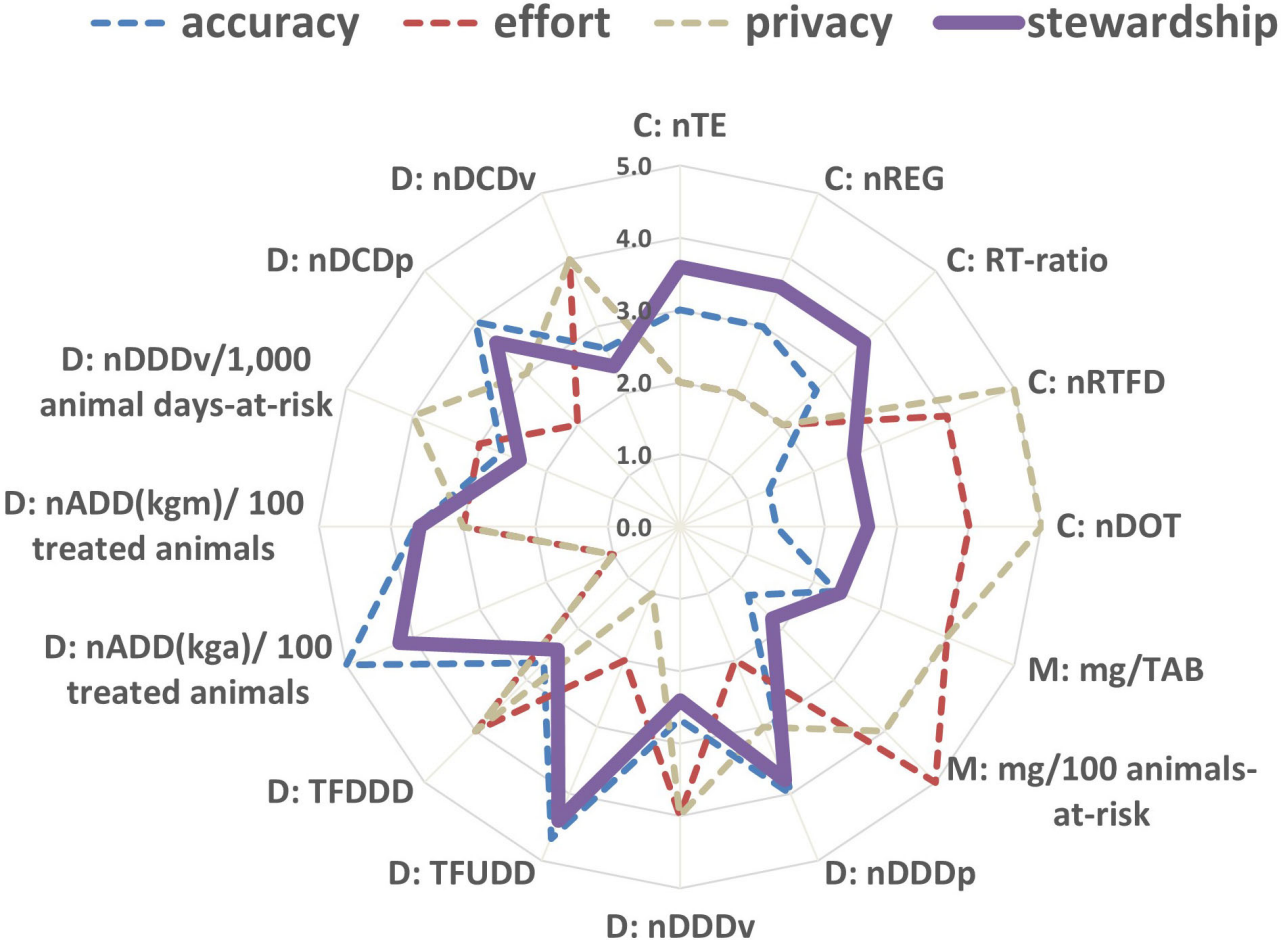
Spider plot showing antimicrobial drug use indicator scores with respect to accuracy (average), effort, privacy and stewardship (average) criteria. Notations ‘C:’, ‘M:’ and ‘D:’ respectively represent the count-based, mass -based, and dose-based indicators defined in **Table 3**.

Heatmaps in **Figures 1**, **2** show scores for the 16 AMU indicators against each individual data requirement- and stewardship information-driven criteria, respectively. A spider plot in **Figure 3** shows how the 16 indicators compare to each other with respect to the effort, privacy, and average scores in accuracy and stewardship criteria. Dose-based indicators generally scored better than count-based and mass-based indicators when accuracy criteria were considered (i.e., ani, ed, and el) (**Figure 1**). Among dose-based indicators, *nADD(kg_a_)/100 treated animals*, *TF_UDD_*, *nDDDp*, *nDCDp*, and *nADD(kg_m_)/100 treated animals* have higher accuracy because they include farm-specific animal body mass and administered dose information. For example, *nADD(kg_a_)/100 treated animals* scored “5” for the three accuracy criteria because it includes actual body mass and dose for each individual treated animal, and consequently, can detect extra-label use. Indicators that use standard dose (*nDDDv*, *TF_DDD_*, *nDDDv/1,000 animal days-at-risk*, and *nDCDv*) or do not include dose information (*nTE*, *nREG*, *RT-ratio*, *nRTFD*, *nDOT*, *mg/TAB*, and *mg/100 animals-at-risk*) cannot detect extra-label use. *TF_UDD_*, which scored “4” in the ed (Exposure data) criterion, does not require the actual dose for each animal but uses the median/mean used daily dose (UDD) administered for an animal production category. Mass-based indicators (*mg/TAB* and *mg/100 animals-at-risk*) capture information about the mass of antimicrobial used but score low (“2”) in the ed criterion demonstrating the limited value of antimicrobial mass alone in characterizing antimicrobial exposure.

The scores for Privacy concerns (pc) and Ease of data recording and calculation (edr or effort criterion) were similar to each other and negatively correlated with the accuracy criteria (**Figure 3**). Indicators with better scores in accuracy criteria (ani, ed, and el) performed poorly in pc and edr. Accurate indicators need farm-level actual information on AMU and require more data. Farmers may have privacy concerns about whether they should record actual animal and dose information and use that granular information in the calculation of indicators, and it takes time to record data needed for all equation terms. In contrast, indicators that include standard animal body mass or defined dose are less accurate but, with a few exceptions, score better in pc and edr because they use existing standard values instead of farm-specific data, which eases the process of recording data and alleviates privacy concerns since they reveal less about the farm practices or herd health. Indicators with standard parameters do not require a prior calculation for the dose terms, such as *UDD* and *ADD_i_*, which eases their calculation.

Based on the scores for data requirements (**Figure 1**), *nADD(kg_m_)/100 treated animals* generally performs well in all criteria (score range: 3-4). On the other hand, *TF_UDD_* and *nADD(kg_a_)/100 treated animals* have the highest accuracy, while *nRTFD*, *nDOT*, and mass-based indicators perform well in pc and edr.

As for the stewardship-driven criteria, all indicators except for *RT-ratio* have a term describing the treated or total animal population in an animal production category that changes over time, so they score well in the population trend criterion (tp) (**Figure 2**). The highest scores for *nTE*, *nREG*, and *TF_UDD_* are because they account for both the treated animal and total animal population.

Scores for criteria describing the trends regarding the treated animals (tt) and the antimicrobial substance exposure (texam) showed similar patterns (**Figure 2**) because indicators that use farm-level specific animal information tend to use the farm-specific dose information; that is, the standard animal body mass and defined dose usually appear simultaneously in indicators. Indicators *nDDDp*, *TF_UDD_*, *nDCDp*, *nADD(kg_a_)/100 treated animals*, and *nADD(kg_m_)/100 treated animals* perform well in these two criteria because they use farm-specific animal body mass and doses, which allows observation of potential changes over time. Since *TF_UDD_* and *nADD(kg_a_)/100 treated animals* use individual animal-level information, they get the best scores in these two criteria. However, standard parameters negatively influence the indicators’ ability to monitor trends because they are constant and cannot reflect the changes in animal body mass and/or dose.

A key goal for an antimicrobial stewardship program is to promote shorter durations of antimicrobial therapy when clinically appropriate (Yarrington and Moehring, 2019). The length of antimicrobial treatments differs over time based on many factors, such as the type and severity of the diseases, and the type and effectiveness of the antimicrobial drugs. Therefore, the ability to follow trends in the length of the received antimicrobial treatment (texle) is essential to understanding herd health and informing antimicrobial stewardship. The length of the received antimicrobial treatment (*DOT*) is directly implemented in *nDOT*, *nDDDp*, *TF_UDD_*, and the two *ADD*-based indicators and is accounted for via regimens in *nTE*, *nREG*, *RT-ratio*, and *nRTFD*, resulting in their overall better scores in texle (**Figure 2**). For example, *nDOT* scored a “5” in the texle criterion because it directly provides information on the length of AMU exposure, while it received a poor score (“1”) in the Exposure data (ed) criterion, in the data requirement category, since it captures no information about the actual amount (mass or dose) of antimicrobial administrated in the treatment of an individual animal (according to the definition of the ed criterion in **Table 4**).The remaining indicators not mentioned in this paragraph cannot track treatment length, which results in their poor scores.

Indicators *nTE*, *nREG*, *RT-ratio*, *TF_UDD_*, *nDDDp*, *nDCDp* and the two *nADD*-based indicators either use the population of treated animals or the regimen information for treated animals in the calculation, which strengthens them with respect to the criterion that evaluates the ability to track treatment effort (tte) (**Figure 2**). *RT-ratio* shows the number of antimicrobial regimens by the number of therapeutic events. As such, this indicator proxies the extent of antimicrobial administration per treated disease event, which reflects the level of preference to use antimicrobials to treat disease, and thus it performs best in tte. Only *nTE*, *nREG*, and *TF_UDD_* indicators account for treated animals and the total population size and track AMU for a given population category, thus can, to a limited degree, reflect the disease pressure in the herd.

Indicators *TF_UDD_* and *nADD(kg_a_)/100 treated animals* have the best performance considering the stewardship-driven criteria, as they can track changes in several aspects relevant to antimicrobial stewardship (**Figure 2**). Indicators with better scores in accuracy tended to have better scores in stewardship (**Figure 3**). *RT-ratio* is a unique indicator; although it does not include animal information, it can show the level of preference for AMU to treat disease events and help explore the feasibility of treating disease with less or no antimicrobials. *nRTFD*, *nDOT*, mass-based indicators, and dose-based indicators using standard parameters are relatively less ideal indicators to inform stewardship because they either lack important animal and antimicrobial information or use standard parameters that cannot capture temporal trends.

## 4 Discussion

Dairy farmers are under pressure to reduce AMU in animals to contribute to the fight against AMR within the One Health initiatives. A number of indicators for tracking farm-level AMU have been published, but they are challenging to interpret and compare (Sanders et al., 2020). Therefore, this study aimed to standardize and evaluate multiple existing AMU indicators based on their accuracy, data needs and effort required, privacy concerns, and ability to inform antimicrobial stewardship in order to aid their uptake on U.S. dairy farms. Our main findings are that: (i) standardized variables/parameters in the AMU indicators allow interchangeable and simultaneous calculation of indicators, (ii) accuracy vs. effort and accuracy vs. privacy trade-offs characterize the evaluated AMU indicators, and (iii) the evaluated AMU indicators can only partially inform antimicrobial stewardship. These findings are discussed in the following paragraphs.

### 4.1 Standardized variables and parameters allow interchangeable and simultaneous calculation of AMU indicators

We standardized and streamlined the calculation of existing AMU indicators, contributing to a better understanding, fair comparison, and easier interpretation of AMU indicators. The availability of these derived standardized indicators is expected to aid their use for on-farm AMU monitoring (Sanders et al., 2020). There have been calls for harmonization and clarification of AMU indicators and their calculations (Monitoring and Evaluation of the Global Action Plan on Antimicrobial Resistance; Sanders et al., 2020; Umair et al., 2021). For example, Umair et al. raised a concern that different AMU measurement metrics create difficulties in comparing AMU data from different sources, which stressed the urgency for developing a globally harmonized AMU measurement system (Umair et al., 2021). Interchangeability between standardized indicators will aid interpretation and allow for more flexibility in their use in monitoring AMU. To our knowledge, the standardization of equations and underlying terms for 16 indicators was an unparalleled undertaking for AMU in animals that may provide a template for future AMU indicator harmonization efforts in other animal species and production categories. The simultaneous calculation of multiple indicators provides a more holistic view of AMU on a farm since no single indicator can comprehensively represent all aspects of antimicrobial decisions, which require nuanced, complex clinical decision-making, and dynamic changes over the course of therapy (Yarrington and Moehring, 2019). For example, some veterinarians perceived that *ADD-based* indicators are less intuitive than the treatment duration (Redding et al., 2020). Also, some standard parameters, such as those for the animal body mass, and the dose and duration of treatment, may differ significantly from the actual situation and thus limit the accuracy of indicators calculated from them. Specifically, the recommended doses of drugs containing the same active substance and used for the same animal body mass can vary considerably between countries and even within countries. Therefore, indicators with actual and updated information instead of standard parameters have better operational accuracy (i.e., better capacity to accurately reflect a farm’s AMU) (Collineau et al., 2017; Mills et al., 2018). However, future research is needed to evaluate the 16 standardized AMU indicators in a field study. Additionally, research is needed to develop a dashboard for simultaneous visualization of multiple AMU indicators and their trends, and an assessment of the experience of farmers and veterinarians using it (Taber et al., 2021).

### 4.2 Evaluated AMU indicators are characterized by the accuracy vs. effort and accuracy vs. privacy trade-offs

Accurate indicators for quantifying AMU in dairy cattle are critical for monitoring trends in animal exposure to antimicrobial drugs over time. Accuracy is necessary, though not sufficient, for the indicator’s utility in informing antimicrobial stewardship (**Figure 3**). Also, in the long run, accurate indicators will enable researchers to examine potential associations between AMU and the emergence of AMR determinants and resistant bacteria in cattle (Brault et al., 2019b). Among the 16 AMU indicators evaluated in this study, we consider *TF_UDD_* and *nADD(kg_a_)/100 treated animals* to be the most accurate. However, their accuracy comes with a price in terms of the increased effort required for data collection (**Figure 3**); *nADD(kg_a_)/100 treated animals* requires data about the dose and body mass for each treated animal, the collection of which is not currently feasible on all farms (Brault et al., 2019a). The data needed for these indicators could be simplified (**Table 5**), reducing the required effort, but at a loss of accuracy and utility for informing antimicrobial stewardship. Thus, a farmer will need to balance accuracy and effort in calculating AMU indicators if their goal is to maximize the private benefits from the AMU indicators for their farm.

A recent survey revealed that 69.3% of dairy farmers in the northeastern U.S. would be interested in knowing how AMU on their farm compares to the use of antimicrobials on other comparable dairy farms (Casseri et al., 2022). This suggests that farmers are not just interested in learning-by-doing (through private use of AMU indicators for their farms) but also learning from their social networks, which would require some form of data sharing (ignoring the possibility of free-riding). For shared AMU indicators to be useful to other farmers in the social network, the values of AMU indicators would need to be accompanied by information that describes the context in terms of farm characteristics and practices (e.g., farm size, number of animals and their production categories, herd health and management, AMU data used for calculation of AMU indicators, and even any evidence of AMR on the farm). Such assembled shared database of AMU indicators and contextual data would provide unprecedented opportunities for data-driven innovation of antimicrobial stewardship in dairy farming. However, the requirement for sharing contextual data with AMU indicators, particularly for more accurate indicators that are based on granular antimicrobial and animal data, may raise concerns, such as regarding potential reputational damages, misuse of data, unauthorized use of data, loss of business advantage, or legal liabilities (Wiseman et al., 2019; Linsner et al., 2021). Thus, sharing AMU data among farmers may raise privacy concerns, creating a trade-off between indicator accuracy (utility) and privacy implications. The conflict between privacy and utility is a well-known concept in research about data sharing (Wirth et al., 2021). Accordingly, sharing accurate indicators would require limitations in the amount/type of contextual data shared to provide privacy, while data sharing that protects privacy would decrease the utility of the data (Wirth et al., 2021). To alleviate privacy concerns, several data sharing techniques have been suggested, from combining, perturbing, removing, or summarizing the data in a way that maintains confidentiality, to algorithms for differential privacy and federated learning (Ritchie, 2011; Qian et al., 2022). Based on the privacy concern and accuracy criteria evaluated in this study, farmers may elect to share *nDDDp* or *nDCDp* indicators. These indicators are limited in answering some questions on stewardship, but do not require individual animal information for calculation. Regarding data sharing, recently emerged initiatives work towards setting principles on data privacy, storage, collection, ownership and processing in the agriculture systems globally and in the U.S (Farm Progress; Data Privacy and Use White Paper), as well as providing educational training for farmers so that farmers can advance their skills on intelligent systems and protect their data (Kaur et al., 2022). A recent pilot project in the U.S. has demonstrated the willingness of swine farms to share AMU data (Davies and Singer, 2020). A century-old National Cooperative Dairy Herd Improvement Program (NCDHIP) provides a roadmap on how to develop a system for cooperative data governance and sharing of AMU and AMR data in the dairy sector (Hutchins and Hueth, 2023).

### 4.3 Evaluated indicators can only partially inform antimicrobial stewardship

Antimicrobial stewardship is defined as finding an optimal approach to sustaining animal health, welfare and production, minimizing selection for AMR and preserving antimicrobial efficacy through conscientious oversight (Apley et al., 2018; American Veterinary Medical Association; Antimicrobial Stewardship Guidelines). This may involve reductions in antimicrobials for animals who do not require them or increases for those who need them; notably, a responsible antimicrobial stewardship program cannot, and should not, strive towards “zero” AMU when measuring over large populations of animals (Yarrington and Moehring, 2019). Unfortunately, based on the information that makes up the components of AMU indicators, which focus on the antimicrobials rather than disease information, these indicators cannot capture the nature of the diseases, the accuracy of diagnosis, or the stage of the disease when treated. However, while stewardship has to include knowledge of what disease is being treated and for what purpose, these indicators contain information regarding some aspects of antimicrobial stewardship. In our study, all but one (*RT-ratio*) evaluated AMU indicator can account for changes in the population at risk. Several indicators (*mg/TAB*, *nDDDp, TF_UDD_*, *nADD(kg_a_)/100 treated animals*, *nADD(kg_m_)/100 treated animals*, and *nDCDp*) can monitor changes over time in the body mass of treated animals. All evaluated indicators capture the production category of treated animals. As such, these indicators can inform stewardship since body mass of treated animals, the at-risk population, and animal production category information can potentially be used for comparisons of AMU among animal groups or to track changes in the same group over time (Canadian Integrated Program for Antimicrobial Resistance Surveillance; Brault et al., 2019b). A few indicators (*RT-ratio*, *nTE*, *nREG*) track antimicrobial treatments, and as such can directly inform antimicrobial stewardship, especially when used along with collected antimicrobial amount data (Schrag et al., 2022). This way, AMU can be associated with specific diseases, and actionable insights can be gained about the necessity of AMU. For example, Schrag et al. proposed recording therapeutic events as a proxy for disease incidence on dairy farms. As a result, they showed the frequency of AMU per treatment event on each farm and were able to identify farms with a high AMU (per treatment event); such farms may be interested in learning about stewardship practices on farms with a low AMU per treatment event (Schrag et al., 2022). Additionally, several indicators (*mg/TAB*, *mg/100 animals-at-risk*, *nDDDp*, *nDDDv*, *TF_UDD_*, *TF_DDD_*, *nADD(kg_a_)/100 treated animals*, *nADD(kg_m_)/100 treated animals*, *nDDDvC/1,000 animal days at risk* and *nDCDv*) can monitor changes in the amount of antimicrobial administered (either in terms of mass or dose). Mass-based indicators provide an intuitive interpretation of AMU, are relatively easy to record, and are frequently used for surveillance (Merle and Meyer-Kühling, 2019; Köper et al., 2020; Tiseo et al., 2020). Also, they are suitable for tracking AMU in specific target populations (e.g., same animal species and production type), and focusing on the same active substance and administration route (Collineau et al., 2017). However, mass-based indicators are confounded by drug potency (Jensen et al., 2004; Brault et al., 2019a). Specifically, if we compare the use of two antimicrobial products with different potency, the product with higher potency will have a lower mass of antimicrobial consumption, but that does not necessarily represent a more judicious antimicrobial use. Therefore, when mass-based indicators are used to compare the AMU of drugs with different potency, this comparison can be misleading (Brault et al., 2019a). In addition, drug potency reflected by dose and duration of treatment is necessary to compare treatment effectiveness and selection pressure, which is not available with mass-based indicators (Chauvin et al., 2001). On the other hand, dose-based indicators reflect how antimicrobial drugs are actually used in animals and consequently are better indicators for informing antimicrobial stewardship efforts (Bright-Ponte, 2020). Brault et al. effectively demonstrated the contrast of using mass- and dose-based metrics in a specific case of macrolide use on beef cattle feedlots, where the use of the mass-based metric resulted in the interpretation of less macrolide use than if the dose-based metric was used (Brault et al., 2019b). Their results demonstrated that mass-based indicators should be used in conjunction with dose-based indicators for creating effective stewardship strategies, especially for macrolides and other medically important antimicrobials with relatively low dose/kg rates (More, 2019). This also demonstrates the value of standardized AMU indicators resulting from this study and the value of simultaneous calculation and visualization of multiple AMU indicators discussed above.

The length of therapeutic effect is important to consider in planning stewardship efforts (More, 2019) and to study the association between antimicrobial exposure in animals and subsequent potential selection of AMR organisms in humans, animals or the environment. In our study, the texle criterion focused on indicators’ ability to provide information on the duration of treatment (*DOT*). The DOT was also used to indirectly evaluate the duration of antimicrobial effect (DOE), which is the period that antimicrobials remain active in an animal’s body. As the DOE of some antimicrobials in animals had not been established, using indicators that utilize DOT can serve to indirectly reflect the DOE of different types and doses of antimicrobials (Brault et al., 2019a). The higher scoring indicators were identified among the count-based (*nRTFD* and *nDOT*) and dose-based indicators (*nDDDp*, *TF_UDD_*, and *nADD(kg_a_)/100 treated animals*). This is unsurprising, since regimens and doses intrinsically account for *DOT* in calculation. However, high scoring indicators in this criterion (texle) should be interpreted carefully since *DOT* is affected by how the dose and/or regimen data are collected. Use of standard doses (*DDDv* and *DCDv*) or standard time intervals (*int* and *adjF*), that are taken directly from treatment protocols or prescriptions, are unable to capture deviations from the protocol or prescription (Schrag et al., 2020b). Furthermore, for long-acting antimicrobials, the actual DOE is not always clear (Brault et al., 2019a). Consequently, these indicators may provide an imprecise information about the length of antimicrobial selective pressure.

It is known that AMU exerts selective pressure on commensal microflora and pathogens, increasing the risk of recovery of AMR microorganisms from treated animals during or after the treatment (Catry et al., 2016; European Centre for Disease Prevention and Control (ECDC) et al., 2017; Lhermie et al., 2017). However, as mentioned above, none of the 16 evaluated indicators can quantify antimicrobial selective pressure. Administration of a single drug leads to selective pressure and the potential development of resistance or cross-resistance. Higher doses of antimicrobials and long treatment times intensify the selective pressure (Raymond, 2019). The antimicrobial administration routes, such as oral administration and intravenous injection routes, have different selective pressure effects on resistance (Zhang et al., 2013). Thus, the antimicrobial drug type, dose, treatment time, and administration route can all shape selective pressure and influence the AMR risk. For example, Volkova et al. successfully established a mathematical model to explore the effect of pharmacokinetics and biodegradation of parenterally administered ceftiofur on the dynamics of ceftiofur-resistant commensal enteric *Escherichia coli* in cattle (Volkova et al., 2012). Future research could be directed at building similar models of the relationship between AMU and selective pressure and the AMR risk level on a farm and scaling them up for use as a novel AMU indicator.

While indicators like nTE and RT-ratio can track the treatment effort on a farm, none of the 16 evaluated indicators can reflect the true disease burden (in terms of disease incidence and prevalence) on a farm. That is because these indicators primarily focus on antimicrobial treatments but do not combine AMU with information about disease occurrence in individual animals. Information about the true disease occurrence in an animal would help avoid misuse of antimicrobials and reduce AMU in healthy individuals, and expose the absence of AMU in situations when treatment is indicated (thus protect animal welfare); therefore, information about true disease occurrence would improve antimicrobial stewardship (Scott, 2013; Nielsen et al., 2021). Repeated testing of all or even a representative sample of animals on a farm to determine the true disease occurrence over time for multiple diseases clearly is not feasible. However, advances in precision livestock farming that uses sensors and other technologies to gather data about every animal on a farm and use that data to optimize herd health management and for early disease detection, are expected to fill that gap (Monteiro et al., 2021). These technologies should be investigated as a source of information about the true disease incidence to improve novel AMU indicators and contribute to antimicrobial stewardship.

In this study, we reviewed the literature and selected for standardization and comparison 16 internationally known AMU indicators suitable for monitoring AMU on U.S. dairy farms. In the future, the same standardization and evaluation approach could be applied to other AMU indicators that may have been missed in the current study. Additionally, future studies should apply the derived standardized AMU indicators to data from multiple farms to evaluate their field accuracy and practical utility and to statistically compare indicators to improve understanding of the best approach to using multiple indicators simultaneously. In the absence of established weights for the criteria used in indicator scoring, all criteria were given the same weights, which affected our conclusions. Future research with stakeholders is necessary to determine whether these criteria should have different weights. We acknowledge that AMU indicator scores are authors’, and, thus, by definition, subjective. However, because scoring involved comparisons of the elements of equations (i.e., presence or absence of terms or information in the formula), the room for subjective interpretation of AMU indicators was limited, allowing us to consider the approach sufficiently objective for their scoring.

## 5 Conclusion

Standardizing the definitions and formula of the AMU indicators will facilitate their uptake by farmers and veterinarians while enabling their interchangeability and fair comparison of AMU indicators across farms. Accuracy and data availability are the first factors to consider, particularly because accuracy is necessary to inform stewardship and for analyses of the relationship between AMU and AMR. At the same time, privacy considerations are also crucial for farmers interested in learning stewardship from their social network, because farmers may be reluctant to share indicators based on detailed AMU information. Overall, according to the criteria established in this study, two dose-based indicators (*TF_UDD_* and *nADD(kg_a_)/100 treated animals*) scored best in accuracy and ability to inform stewardship, while two count-based indicators (*nRTFD* and *nDOT*) and a mass-based indicator (*mg/100 animals-at-risk*) performed best in the effort and privacy criteria.

## Data availability statement

The original contributions presented in the study are included in the article/**Supplementary Material**. Further inquiries can be directed to the corresponding author.

## Author contributions

ZL finished the literature review and drafted the first version of the manuscript. ZL, EB and RI built the standardization and scoring approaches together. EB and RI gave their initial comments on the first manuscript version. ZL, EB, DN, and RI contributed to subsequent revisions and approved the final version.
