# Supplementary material for: Standardization and evaluation of indicators for quantifying antimicrobial use on U.S. dairy farms
Source: Front Antibiot. 2023 Aug 14;2:1176817. doi: 10.3389/frabi.2023.1176817 (PMC11731823; doi:10.3389/frabi.2023.1176817)
Supplement: Supplementary file 1 [file DataSheet_1.docx]

Supplementary Material

Standardization and evaluation of indicators for quantifying antimicrobial use on U.S. dairy farms

Zhengyu Lu*, Ece Bulut, Daryl V. Nydam, Renata Ivanek

*** Correspondence:** Corresponding Author: [zl426@cornell.edu](mailto:zl426@cornell.edu)

# Supplementary Tables

**Supplementary Table S1. Definitions of original vs standardized terms (in Tables 1 and 2) used in the calculation of antimicrobial use indicators (Table 3)**

| **Original term notation** | **Original definition** | **Reference** | **Standardized term notation** | **Standardized definition** |
| --- | --- | --- | --- | --- |
| DS | Disease Syndrome | (1) | *d* | Specific treatment indication/disease syndrome (treatment indication) |
| g | The amount of active substance by weight, defined as a standardized regimen term | (1) | *m_R_* | Total mass of an active substance (s) over all administrations (c) administrated as part of a specific single regimen for a disease event (d) in an individual animal (i) (mg) |
| Single administration | Drug product administered at a single restraining event | (1) | *a* | Single administration: Antimicrobial product administered at a single restraining event to an individual animal (*i*). Dataset associated with each individual administration: $a=\{ i,t,r,s,m, d,p,w\}$ (administration) |
| SReg | Administration of an antimicrobial product for a therapeutic purpose targeting a single disease event in an individual animal. Multiple administrations are counted as part of a single regimen when product administrations are consecutive, never resulting in a time gap between administrations of greater than the pre-determined administration interval of 5 days. | (1) | *R* | Standard regimen (course): Recorded antimicrobial product administration(s) for a therapeutic purpose targeting a single disease event (*d*) in an individual animal (*i*). Multiple administrations in an animal (*a_i_*) are counted as part of a single regimen when product administrations are consecutive, never resulting in a time gap between administrations of greater than the pre-determined administration interval of 5 days. Dataset associated with each individual administrated regimen: $R=\{ i,t_{first}, t_{last}, r,s,m, d,p,w,c_{R},int,adjF\}$ (regimen) |
| REG | Summation of the number of standard regimens (SReg) recorded in the treatment record. | (2) | *R_T_* | Total number of all standard regimens (*R*) administered on a farm *f* during a period of time *T*. Can be calculated overall ($R_{T}$), or subset for a specific production category (p), active substance (s), route of administration (r), disease (d), or their combination. (regimen) |
| Administered dose | The administered dose to all treated animals | (3) | *AD_i_* | The actual dose (*m_i_*/*w_i_*) of an active substance (*s*) in a single antimicrobial administration for a therapeutic purpose targeting a single disease event (*d*) in an individual animal (*i*) (mg active substance/kg animal) |
| Administered dose | The administered dose to all treated animals | (3) | *AD_m_* | Prescribed or mean dose of an active substance (*s*) in a single antimicrobial administration for a therapeutic purpose targeting a single disease event (*d*) in an individual animal (*i*) (mg active substance/kg animal) |
| ADD_kg_ | Actual daily dose of antimicrobial drug used | (3) | *ADD_i_* | Actual daily dose for an active substance (*s*) in a single antimicrobial administration for a therapeutic purpose targeting a single disease event (*d*) in an individual animal (*i*) (mg active substance/kg animal/day) |
| ADD | Mg/day dosage for an animal of standard weight | (3) | *ADD_m_* | Prescribed or mean daily dose for an active substance (*s*) in a single antimicrobial administration for a therapeutic purpose targeting a single disease event (*d*) in an individual animal (*i*) (mg active substance/kg animal/day) |
| UDD | The Used Daily Dose, defined as the actual administered dose per actual kg animal per day | (4) | *UDD* | Median (preferred) or mean of actual used daily doses administered per day as part of a regimen per actual kg of animal body mass at the time of treatment (wR) on farm f during a time period T (mg active substance/kg animal/day) |
| DDDstudy | Study-defined daily doses, calculated using a daily dose defined at the study level | (2) | *DDDp* | Study-defined daily dose that is specific for the population under study (mg active substance/kg animal/day) |
| DDD_vet_ | The defined daily dose for animals, defined as the average of all observations of daily doses by species, substance and form, published by European Surveillance of Veterinary Antimicrobial Consumption (ESVAC) or Government of Canada | (5,6) | *DDDv* | Standard defined daily dose by the European Surveillance of Veterinary Antimicrobial Consumption or Government of Canada (mg active substance/kg animal/day) |
| DCDstudy | Study-defined course doses, calculated using a course dose defined at the study level | (2) | *DCDp* | Study-defined course dose that is specific for the population under study (mg active substance/kg animal/course) |
| DCD_vet_ | The defined course dose for animals, defined as the assumed average dose per kg animal per species per treatment course | (5,6) | *DCDv* | Standard defined course dose proposed by European Surveillance of Veterinary Antimicrobial Consumption or Government of Canada (mg active substance/kg animal/course) |
| ANIMALS | The number of animals exposed. This is a unitless count. | (1) | *n_wk,p_* | Number of animals of a given production category (*p*) present on a farm *f* in a given week (*wk*) (animal) |
| ANIMALS | The number of animals exposed. This is a unitless count. | (1) | *n_wk_* | Number of animals of any production category present on a farm *f* in a given week (*wk*) (animal) |
| ANIMALS | The number of animals exposed. This is a unitless count. | (1) | $\bar{N_{p}}$ | Average number of animals of a given production category (*p*) on a farm *f* (or average farm inventory of a given production category (*p*)) during a time period *T* (animal) |
| Animal weight at treatment | Weight of the treated animal | (4) | *w_i_* | Body mass of an individually treated animal at the time of antimicrobial product administration (can be measured or estimated from animal age at the time of treatment using growth charts) (kg) |
| Standard weight | The mean weight of cattle at time of exposure to any antimicrobial drug on a farm | (3) | *w_f,p_* | Farm *f* specific average body mass (or farm-specific standard body mass) for the production category (*p*) of a treated animal at the time of antimicrobial product administration. Can be obtained from historical farm records or by measuring a representative subset of animals (kg) |
| Standard animal weight | The theoretical weight of an animal at the likely time of treatment | (5,7) | *w_p_* | Standard average body mass for the production category (*p*) of a treated animal at the time of drug product administration (kg) |
| Administration interval | Interval between administrations | (1) | *int* | Interval between administrations within a single regimen that is less than 24h (day) |
| *adjFactor* | Time interval between doses, used when interval is ≥ 1 day | (2) | *adjF* | Adjustment factor for long-acting antimicrobial products, for which single administration provides > 1 day of therapy. Can be the time interval between administrations or estimated duration of antimicrobial effect (unitless) |
| aDOT | Adjusted days of therapy | (2) | *DOT* | Duration of treatment. Depending on antimicrobial product used, *DOT* is expressed as: *cDOT*: Count of calendar days on which treatment was administered as part of a single regimen, used for antimicrobials administered in intervals ≤1 day; *aDOT*: Adjusted length of therapy for a single regimen used for a long-acting antimicrobial product or product administered in intervals > 1 day. (day) |
| cDOT | Calendar days of therapy | (2) | *DOT* | Duration of treatment. Depending on antimicrobial product used, *DOT* is expressed as: *cDOT*: Count of calendar days on which treatment was administered as part of a single regimen, used for antimicrobials administered in intervals ≤1 day; *aDOT*: Adjusted length of therapy for a single regimen used for a long-acting antimicrobial product or product administered in intervals > 1 day. (day) |
| DOE | Duration of effect of an antimicrobial drug | (3) | *DOT* | Duration of treatment. Depending on antimicrobial product used, *DOT* is expressed as: *cDOT*: Count of calendar days on which treatment was administered as part of a single regimen, used for antimicrobials administered in intervals ≤1 day; *aDOT*: Adjusted length of therapy for a single regimen used for a long-acting antimicrobial product or product administered in intervals > 1 day. (day) |
| Animal days at risk | Also known as standard-animals at risk, accounts for the inter-species variations in live animal biomass and duration of the grow-out or observation period. It is the average days at risk or lifespan of the animal | (5) | *ADR* | Average days at risk: an average number of days individual animals of production category *p* are present on farm *f* (days) |
| TF | Regimen time frame, defined as the number of calendar days between the first and last administration | (1) | *cfl_R_* | The number of calendar days between the first and last administration of a regimen to an animal (*i*) (day) |

**Supplementary Table S2. Definition of main abbreviations and acronyms used in derivation and evaluation of antimicrobial use indicators in alphabetical order.**

| **Abbreviation** | **Definition** | **Described in** |
| --- | --- | --- |
| *ADD* | Animal daily dose | Introduction |
| *adjF* | Adjustment factor for long-acting antimicrobial products | Table 1 |
| *ADR* | Average days at risk | Table 1 |
| *ani* | Scoring criterion: Animal information | Table 4 |
| *cfl_R_* | Number of calendar days between the first and last administration in a regimen | Table 2 |
| *DCD* | Defined course dose | Introduction |
| *DCDv* | Defined course dose by the European Surveillance of Veterinary Antimicrobial Consumption (ESVAC) | Table 1 |
| *DDD* | Defined daily dose | Introduction |
| *DDDv* | Defined daily dose by ESVAC | Table 1 |
| *DOE* | Duration of antimicrobial effect | Discussion |
| *DOT* | Duration of treatment | Table 2 |
| *ed* | Scoring criterion: Exposure data | Table 4 |
| *edr* | Scoring criterion: Ease of data recording and calculation | Table 4 |
| *el* | Scoring criterion: Extra-label use | Table 4 |
| *ESVAC* | European Surveillance of Veterinary Antimicrobial Consumption | Introduction |
| *Int* | Interval between administrations within a single regimen that is less than 24h | Table 1 |
| *K_p_* | Total number of treated animals in a production category | Table 2 |
| *m_p_* | Total mass of all active substances used in an animal production category on farm *f* during a period of time *T* | Table 2 |
| *m_p,s_* | Total mass of an active substance used in an animal production category on farm *f* during a period of time *T* | Table 2 |
| *m_R_* | Total mass of an active substance over all administrations administrated as part of a specific single regimen in an individual animal | Table 2 |
| $\bar{m_{R}}$ | Mean mass of an active substance over all instances of application of a specific regimen administrated during a period of time *T* | Table 2 |
| *nADD(kg_a_)/100 treated animals* | Number of animal daily doses per 100 treated animals that use the individual animal AMU and body mass information | Table 3 |
| *nADD(kg_m_)/100 treated animals* | Number of animal daily doses per 100 treated animals that use the average animal AMU and body mass information | Table 3 |
| *nDCDp* | Number of study defined course doses | Table 3 |
| *nDCDv* | Number of standard defined course doses | Table 3 |
| *nDDDp* | Number of study defined daily doses | Table 3 |
| *nDDDv* | Number of standard defined daily doses | Table 3 |
| *nDDDv/1,000 animal days-at-risk* | Number of Canadian-defined daily doses per 1,000 animal days-at-risk | Table 3 |
| *nDOT* | Total length of all therapies | Table 3 |
| $\bar{N_{p}}$ | Average number of animals of a given production category on a farm f (or average farm inventory of a given production category) during a time period *T* | Table2 |
| *nREG* | Number of standard regimens | Table 3 |
| *nRTFD* | Number of regimen time frame days | Table 3 |
| *nTE* | Number of therapeutic events | Table 3 |
| *pc* | Scoring criterion: Privacy concerns | Table 4 |
| *PCU* | Population correction unit | Introduction |
| *RT-ratio* | Antimicrobial regimen to therapy ratio | Table 3 |
| *sp* | Scoring criterion: Standard parameters | Table 4 |
| *SReg* | Standard regimen | Introduction |
| *TAB* | Target animal biomass | Introduction |
| *texam* | Scoring criterion: Trends over time regarding exposure: antimicrobial substance | Table 4 |
| *texle* | Scoring criterion: Length of treatments | Table 4 |
| *TF_DDD_* | Treatment frequency calculated with the standard defined daily dose | Table 3 |
| *TF_UDD_* | Treatment frequency calculated with the used daily dose | Table 3 |
| *tp* | Scoring criterion: Trends over time regarding population at risk | Table 4 |
| *tt* | Scoring criterion: Trends over time regarding treated animals | Table 4 |
| *tte* | Scoring criterion: Trends over time regarding treatment effort | Table 4 |
| *UDD* | Used daily dose | Table 2 |
| *w_f,p_* | Average body mass for animals of the treated animal production category on a specific farm | Table 1 |
| *w_i_* | Measured or estimated body mass of an individual treated animal | Table 1 |
| *w_p_* | Standard average body mass for the treated animal production category | Table 1 |

**Supplementary Table S3. Reasoning regarding scoring of antimicrobial use indicators for data requirement-driven criteria**

| ***Criteria***  ***Indicator*** | Animal Information (ani) | Exposure data (ed) | Extra label use (el) | Standardized parameters (sp) | Privacy concerns (pc) | Ease of data recording and calculation (edr) |
| --- | --- | --- | --- | --- | --- | --- |
| ***nTE*** | 2-Actual average animal population size and population of treated animals information in the regimen. No animal body mass | 3-No mass of antimicrobial or dose information but includes regimens, which record each treatment | 4-No dose information but calculated from regimens that record each treatment | 1-No standard parameters | 2-Requires times of therapeutic events, which can reflect disease burden | 2-Calculation is easy by regimens. It requires plenty of information. |
| ***nREG*** | 2-Actual average animal population size and population of treated animals information in the regimen. No animal body mass | 3-No mass of antimicrobial or dose information but includes regimens, which record each treatment | 4-No dose information but calculated from regimens that record each treatment | 1-No standard parameters | 2-Requires times of regimens, which can reflect the frequency of antimicrobial use | 2-Calculation is easy by regimens. It requires plenty of information. |
| ***RT-ratio*** | 1-No animal population and body mass information | 3-No mass of antimicrobial or dose information but includes regimens, which record each treatment | 4-No dose information but calculated from regimens that record each treatment | 1-No standard parameters | 2-Requires times of therapeutic events and regimens, which can reflect the frequency of antimicrobial use | 2-Calculation is easy by regimens. It requires plenty of information. |
| ***nRTFD*** | 2-Actual average animal population size and population of treated animals information in the regimen. No animal body mass | 1-No mass of antimicrobial or dose information | 1-No dose information, extra-label use cannot be detected | 1-No standard parameters | 5-Regimen time frame days and animal population are needed only | 4-One step calculation. Although it is based on regimen, time frame data is enough for calculation. |
| ***nDOT*** | 2-Actual average animal population size and population of treated animals information in the regimen. No animal body mass | 1-No mass of antimicrobial or dose information | 1-No dose information, extra-label use cannot be detected | 1-No standard parameters | 5-Duration of treatments and animal population are needed only | 4-Two step calculation. Requires calculation of *DOT* |
| ***mg/TAB*** | 4-Actual average population size and farm estimated average body mass | 2-Mass of antimicrobials | 1-No dose information, extra-label use cannot be detected | 4- Can use standard body mass or estimated mean body mass | 4-Mass of active substances, animal body mass and animal population are needed only, may be calculated by estimated animal body mass | 4-Two step calculation since estimated mean body mass may be required |
| ***mg/100 animals-at-risk*** | 1-Actual average animal population size without body mass information | 2-Mass of antimicrobials | 1-No dose information, extra-label use cannot be detected | 1-No standard parameters | 4- Mass of active substances and animal population are needed only | 5-One step calculation. |
| ***nDDDp*** | 4-Actual average population size and farm estimated average body mass | 4-Actual mass of antimicrobials and actual mean dose for treated animals | 4-Uses regimens to calculate the mean mass of an active substance. The process of calculating the mean mass of active substance may find extra-label use | 3-Can use estimated mean body mass | 3-Dose information, mass of active substances, animal body mass and animal population are needed | 2-Two-step calculation. However, requires calculation of *DDDp*, *DOT*, and estimated mean body mass |
| ***nDDDv*** | 3-Actual average population size and standard animal body mass | 3-Actual mass of antimicrobials and standard dose for treated animals | 2-Uses the standard dose for treated animals. Extra label use would not be detected. | 5-Uses standard mean body mass and standard dose | 4-Mass of active substances and animal population are needed | 4-One step calculation. Requires mass of antimicrobials and average animal population size |
| ***TF_UDD_*** | 5-Actual number of treated animals and actual animal body mass | 4-Actual mass of antimicrobials and actual mean dose for treated animals | 5-Uses the total mass of an active substance for an individual animal | 1-No standard parameters | 1-Requires dose information for each animal, mass of active substances for each animal, number of treated animals, animal population, and individual animal body mass | 2- Two-step calculation. Requires recording mass of antimicrobials and body mass for each animal, but can also use estimated mean body mass and mean mass of an active substance to calculate UDD |
| ***TF_DDD_*** | 3-Actual average population size and standard animal body mass | 3-Actual mass of antimicrobials and standard dose for treated animals | 2-Uses the standard dose for treated animals. Extra label use would not be detected | 5-Uses standard mean body mass and standard dose | 4-Requires mass of active substances, animal population data and standard dose information | 4-One step calculation. Requires mass of antimicrobials and average animal population size |
| ***nADD(kg_a_)/ 100 treated animals*** | 5-Actual number of treated animals and actual animal body mass | 5-Actual mass of antimicrobials and actual dose for each treated animal | 5-Has the actual dose information for an individual animal | 2-*adjF* and *int* for each drug are standard | 1-Requires dose information for each animal, mass of active substances for each animal, number of treated animals, animal population, and individual animal body mass | 1-Multiple step calculation. Need to record mass of antimicrobials, actual dose and body mass for each animal, animal population and *DOT* |
| ***nADD(kg_m_)/ 100 treated animals*** | 4-Actual number of treated animals and farm estimated average body mass | 4-Actual mass of antimicrobials and actual mean dose or prescribed dose for treated animals | 3-Uses the mean dose or prescribed dose for treated animals. Extra label use may be detected if the mean dose is used | 4-Prescribed dose, standard *adjF* and estimated mean body mass | 3-Requires mass of active substances, estimated mean animal body mass, prescribed or mean dose and number of treated animals | 3-Two step calculation. Need to use the mass of antimicrobials and estimated mean animal body mass and number of treated animals |
| ***nDDDv/1,000 animal days-at-risk*** | 3-Actual average population size and standard animal body mass | 3-Actual mass of antimicrobials and standard dose for treated animals | 2-Uses the standard dose for treated animals. Extra label use would not be detected | 5-Uses standard mean body mass and standard dose | 4-Requires mass of active substances, animal population and standard dose information | 3-One step calculation. Requires mass of active substances, average animal population size, and average days at risk |
| ***nDCDp*** | 4-Actual average population size and farm estimated average body mass | 4-Actual mass of antimicrobials and actual mean dose for treated animals | 4-Uses regimens to calculate the mean mass of an active substance. The process of calculating the mean mass of active substance may detect extra-label use | 3-Can use estimated mean body mass | 3-Requires mass of active substances, animal body mass, animal population and dose information | 2- Two-step calculation. Requires calculation of *DCDp* from the mean mass of antimicrobials and estimated mean body mass |
| ***nDCDv*** | 3-Actual average population size and standard animal body mass | 3-Actual mass of antimicrobials and standard dose for treated animals | 2-Uses the standard dose for treated animals. Extra label use would not be detected | 5-Uses standard mean body mass and standard dose | 4-Requires mass of active substances, standard dose information, and animal population | 4-One step calculation. Requires mass of antimicrobials, average animal population size and standard dose |

**Supplementary Table S4. Reasoning regarding scoring of antimicrobial use indicators for stewardship-driven criteria**

| ***Criteria***  ***Indicator*** | Trends over time regarding treated animals (tt) | Trends over time regarding population at risk (tp) | Trends over time regarding treatment effort (tte) | Trends over time regarding exposure: antimicrobial substance (texam) | Trends over time regarding exposure: length (texle) |
| --- | --- | --- | --- | --- | --- |
| ***nTE*** | 3- Therapeutic event (*TE*) consists of regimens, which contain changing individual characteristics and treatment indications but does not reflect these changes in the indicator | 5-Uses changing number of animals of a given production category and recorded treated animal population in the regimen | 4-The *TE* is a proxy for the treated disease events in an animal population. No indication about the proportion of diseased animals treated | 3-Not informative as *nREG*, but regimen is part of *TE* which includes antimicrobial mass and dose information | 3-Has information about the length of treatment through actual dose information but does not reflect the change in the indicator |
| ***nREG*** | 3-Standard regimen contains changing individual characteristics and treatment indications but does not reflect these changes in the indicator | 5-Uses changing number of animals of a given production category and recorded treated animal population in the regimen | 3-Can show the number of regimens in the animal population, not informative as *TE* but can reflect information about treated disease events. No indication about the proportion of diseased animals treated | 4-Reflects changes in administrated antimicrobials through regimens | 3-Has information about the length of treatment through actual dose information but does not reflect the change in the indicator |
| ***RT-ratio*** | 3-Standard regimen contains changing individual characteristics and treatment indications but does not reflect these changes in the indicator | 2-Recorded treated animal population in the regimen, but does not reflect the population in the indicator | 5-Contains *TE* information along with the number of standard regimens used for a disease event, reflecting the level of preference to use antibiotics to treat disease | 5-Reflects changes in administrated antimicrobials through regimens, and it can also detect over extensive antimicrobial use | 3-Has information about the length of treatment through actual dose information but does not reflect the change in the indicator |
| ***nRTFD*** | 1-Does not include animal information | 4-Uses changing number of animals of a given production category | 2-Information about which animals receive treatment | 1-No information about changes in administrated antimicrobials | 5-Directly provides information on the length of treatment |
| ***nDOT*** | 1-Does not include animal information | 4-Uses changing number of animals of a given production category | 2-Information about which animals receive treatment | 1-No information about changes in administrated antimicrobials | 5-Directly provides information on the length of treatment |
| ***mg/TAB*** | 4-Uses estimated average body mass for each production category, which can change (unless constant standard body mass is used) | 4-Uses changing number of animals of a given production category | 1-Does not have treated animal information | 2-Reflects changes in the mass of active substances | 1-Indicator does not have timing variable |
| ***mg/100 animals-at-risk*** | 1-Does not include animal information | 4-Uses changing number of animals of a given production category | 1-Does not have treated animal information | 2-Reflects changes in the mass of active substances | 1-Indicator does not have timing variable |
| ***nDDDp*** | 4-Uses estimated average body mass for each production category, which can reflect change | 4-Uses changing number of animals of a given production category | 3-Has the information about used doses for treated animals | 4-Reflects changes in the mass of active substances and mean mass of an active substance over recorded regimens | 4-Has information about the length of treatment in calculation |
| ***nDDDv*** | 2-Uses constant standard body mass for each production category and thus can’t reflect change | 4-Uses changing number of animals of a given production category | 1-Does not have treated animal information | 3-Reflects changes in the mass of active substances and has standard dose | 2-Has information about the length of treatment through standard doses |
| ***TF_UDD_*** | 5-Uses actual animal body mass, which changes over time | 5-Uses changing number of animals of a given production category and treated animal population | 3-The used daily dose (*UDD*) is calculated for each treated animal but the treatment frequency provides no indication about the proportion of diseased animals treated | 5-Reflects changes in the mass of active substances and dose for an individual animal | 4-Has information about the length of treatment in calculation |
| ***TF_DDD_*** | 2-Uses constant standard body mass for each production category and thus can’t reflect changes | 4-Uses changing number of animals of a given production category | 1- Does not have treated animal information | 3-Reflects changes in the mass of active substances and has standard dose | 2-Has information about the length of treatment through standard doses |
| ***nADD(kg_a_)/ 100 treated animals*** | 5-Uses actual animal body mass, which changes over time | 4-Uses changing number of treated animals of a given production category | 3-Has total treated animal population but no indication about the population to estimate proportion of diseased animals treated | 5-Reflects changes in the mass of active substances and dose for each individual animal | 4-Has information about the length of treatment in calculation for each treatment |
| ***nADD(kg_m_)/ 100 treated animals*** | 4-Uses estimated average body mass for each production category, which can change | 4-Uses changing number of treated animals of a given production category | 3-Has total treated animal population but no information about the proportion of diseased animals treated | 4-Reflects changes in the mass of active substances and mean dose | 3-Has information about the length of treatment through actual prescribed or mean dose information |
| ***nDDDv/1,000 animal days-at-risk*** | 2-Uses constant standard body mass for each production category and thus can’t reflect changes | 4-Uses changing number of animals of a given production category | 1- Does not have sick animal information | 3- Reflects changes in the specific mass of active substance and has standard dose | 2-Has information about the length of treatment through standard doses |
| ***nDCDp*** | 4-Uses estimated average body mass for each production category, which can change | 4-Uses changing number of animals of a given production category | 3-Have the information of mean regimens for treated animals | 4-Reflects changes in the mass of specific active substance and mean mass of an active substance over recorded regimens | 3-Has information about the length of treatment through actual dose information |
| ***nDCDv*** | 2- Uses constant standard body mass for each production category and thus can’t reflect changes | 4-Uses changing number of animals of a given production category | 1-Does not have treated animal information | 3-Reflects changes in the mass of active substances and has standard dose | 2-Has information about the length of treatment through standard doses |

# Supplementary Data

**Supplementary Data 1. Illustration of calculation of farm-level antimicrobial use indicators on a hypothetical dairy farm**

Please find the dataset at the following link: <https://github.com/IvanekLab/AMU-indicators>

**References:**

1. Schrag NFD, Apley MD, Godden SM, Lubbers BV, Singer RS. Antimicrobial use quantification in adult dairy cows – Part 1 – Standardized regimens as a method for describing antimicrobial use. Zoonoses and Public Health. 2020;67(S1):51–68.

2. Schrag NFD, Apley MD, Godden SM, Singer RS, Lubbers BV. Antimicrobial use quantification in adult dairy cows – Part 2 – Developing a foundation for pharmacoepidemiology by comparing measurement methods. Zoonoses and Public Health. 2020;67(S1):69–81.

3. Brault SA, Hannon SJ, Gow SP, Otto SJG, Booker CW, Morley PS. Calculation of Antimicrobial Use Indicators in Beef Feedlots—Effects of Choice of Metric and Standardized Values. Front Vet Sci. 2019 Oct 9;6:330.

4. Kasabova S, Hartmann M, Werner N, Käsbohrer A, Kreienbrock L. Used Daily Dose vs. Defined Daily Dose—Contrasting Two Different Methods to Measure Antibiotic Consumption at the Farm Level. Frontiers in Veterinary Science [Internet]. 2019 [cited 2022 Dec 15];6. Available from: https://www.frontiersin.org/articles/10.3389/fvets.2019.00116

5. Canadian Integrated Program for Antimicrobial Resistance Surveillance (CIPARS) 2018: Design and Methods [Internet]. [cited 2022 Dec 21]. Available from: https://www.canada.ca/content/dam/phac-aspc/documents/services/surveillance/canadian-integrated-program-antimicrobial-resistance-surveillance-cipars/cipars-reports/2018-annual-report-design-methods/2018-annual-report-design-methods.pdf

6. Defined daily doses for animals (DDDvet) and defin.pdf [Internet]. [cited 2023 Jan 22]. Available from: https://www.ema.europa.eu/en/documents/other/defined-daily-doses-animals-dddvet-defined-course-doses-animals-dcdvet-european-surveillance_en.pdf

7. Revised ESVAC reflection paper on collecting data .pdf [Internet]. [cited 2023 Jan 22]. Available from: https://www.ema.europa.eu/en/documents/scientific-guideline/revised-european-surveillance-veterinary-antimicrobial-consumption-esvac-reflection-paper-collecting_en.pdf
